# Do the frail experience more adverse events from intensive blood pressure control? A 2-year prospective study in the Irish Longitudinal Study on Ageing (TILDA)

**DOI:** 10.1016/j.eclinm.2022.101304

**Published:** 2022-02-19

**Authors:** Patrick O'Donoghue, Aisling M. O'Halloran, Rose Anne Kenny, Roman Romero-Ortuno

**Affiliations:** a The Irish Longitudinal Study on Ageing, Trinity College Dublin, Dublin, Ireland; b School of Medicine, Trinity College Dublin, Dublin, Ireland; c Discipline of Medical Gerontology, St. James's Hospital, Mercer's Institute for Successful Ageing, James's Street, Dublin 8, Dublin, Ireland

## Abstract

**Background:**

The 2018 European Society of Cardiology/European Society of Hypertension (ESC/ESH) guidelines for management of hypertension in adults aged ≥65 years recommend a blood pressure (BP) treatment target of 130–139/70–79 mmHg if tolerated. Randomised controlled trials have advocated for lower BP, but this may have adverse outcomes in the frail. Yet, definitions of frailty vary.

**Methods:**

Using a prospective, observational study design, we compared two frailty classifications in their ability to predict short-term adverse outcomes associated with intensive BP control (<130/70 mmHg) in The Irish Longitudinal Study on Ageing (TILDA). Data from participants aged ≥65 treated for hypertension in Wave 1 (W1) between October 2009 and June 2011 were analysed. Frailty was identified by Frailty Phenotype (FP) and the Clinical Frailty Scale (CFS). We formulated 8 participant groups based on frailty-BP combinations. Outcomes at wave 2 (W2) in 2012–2013 were analysed with adjusted binary logistic regression models.

**Findings:**

Of 1920 W1 participants aged ≥65 and treated for hypertension, 1229 had full BP/FP and 1282 BP/CFS data. While the FP only identified risk of hospitalisation associated with intensive BP treatment, intensively treated frail-by-CFS participants had no increased or decreased risk of adverse outcomes, but those treated above the target had a higher risk of falls/fractures. In the non-frail by FP, intensive blood pressure treatment was associated with reduced risk of falls/fractures.

**Interpretation:**

Different frailty classifications may have different prognostic implications for the purpose of the application of hypertension management guidelines. Our study had limited power due to low frailty prevalences, so further research is needed. Guidelines should specify the recommended frailty identification method/s. In the frail, therapy personalisation is needed.

**Research in context**

**Evidence before this study**

The 2018 European Society of Cardiology/European Society of Hypertension guidelines for management of hypertension in adults aged ≥65 years recommend a blood pressure (BP) treatment target of 130–139/70–79 mmHg if tolerated. Randomised controlled trials have advocated for lower BP, but this may have adverse outcomes in the frail. Observational studies, in the main, have advocated for lenient higher BP targets in frail older adults. The definitions of frailty also vary with different operationalisations of frailty such as the frailty phenotype (FP) and Clinical Frailty Scale (CFS). Therefore, the evidence for hypertension treatment targets in the frail are conflicting. To appraise the current evidence, we searched PubMed to identify articles published from January 1980 to October 2021 using the key search words “hypertension”, “frailty” and “older adult”.

**Added value of this study**

This prospective, observational study of community-dwelling older (aged ≥ 65 years) adults from the Wave 1 (2009–2011) of The Irish Longitudinal Study on Ageing (TILDA) identified 1920 participants at Wave 1 who were aged ≥65 and treated for hypertension, of which 1229 had full BP/FP and 1282 BP/CFS data. Intensive BP treatment was defined as BP <130/70 mmHg. At Wave 2 (2012–2013) follow up, the FP only identified an increased risk of hospitalisation associated with intensive BP treatment, while intensively treated frail-by-CFS participants had no increased or decreased risk of any adverse outcomes, but those frail-by-CFS treated above the BP target had a higher risk of falls/fractures. In the non-frail by FP, intensive blood pressure treatment was associated with a reduced risk of falls/fractures. Our study highlights some gaps in the evidence for treating hypertension in the frail.

**Implications of all the available evidence**

Different frailty classifications may have different prognostic implications for the purpose of the application of hypertension management guidelines. Guidelines should specify the recommended frailty identification methods when defining frailty. In the frail, therapy individualisation is warranted. Further research in clinical populations that include participants from throughout the frailty spectrum is required to help evaluate this complex clinical question.

Alt-text: Unlabelled box

## Introduction

Hypertension is a proven risk factor globally for cardiovascular morbidity including ischaemic heart disease, heart failure, stroke and cardiovascular mortality.^1^ In epidemiological studies, long-term treatment of hypertension has been shown to reduce both cardiovascular mortality and all-cause mortality.^2^ However, the management of older adults who have hypertension is not clear-cut, as cohort studies have suggested that the cardiovascular benefits of treatment can be offset by the increased risk of adverse events from anti-hypertensive medication including hypotension, falls and associated injuries.^3^

Indeed, for older adults with hypertension, frailty may add an extra layer of complexity to managing their cardiovascular risks. Frailty has been defined as a state of dysregulation of multiple physiological systems resulting in increased vulnerability to stressors.^4^ Frailty is associated with reduced physiological regulation of various organ systems including blood pressure (BP) homeostasis.^5^ The clinical characterisation of different BP levels in older people living with frailty is mixed in the literature. In the Lausanne 65+ population-based cohort, frailty was associated with a substantially lower BP compared to the non-frail.^6^ However, it has been argued that BP levels in frail older people cannot be easily dichotomised as either low or high given the heterogeneity of this population.^7^

Theoretically and clinically intuitively, the reduced physiological reserve and increased vulnerability associated with a frailty state may confer a greater risk of adverse events from excessive BP-lowering treatment, and some observational studies have contributed supportive evidence in this regard. In a cohort study, Odden et al. used slow gait speed as a marker of frailty and found that those with slow gait speed and hypertension did not have an increased risk of cardiovascular mortality.^8^ In an observational study of more than 415,980 primary care patients aged 75 years or more, Masoli et al. showed that a BP of <130/80 mmHg was associated with excess mortality, while hypertension was not associated with mortality in those aged 75 to 84 years with moderate to severe frailty, or in all aged 85 years or over.^10^ In another cohort study, Ravindrarajah et al. reported that intensive systolic BP (SBP) control of <120 mmHg in patients aged 80 years or more living with frailty was associated with increased all-cause mortality compared to those treated to an SBP between 120 and 139 mmHg or 140–159 mmHg.^11^

Observational studies have generally indicated that lower BP in frail older adults may be associated with negative health outcomes, in particular mortality. However, evidence from randomised controlled trials has been contradictive.^12^ SPRINT showed that in adults aged 75 or more years, treating SBP to a target of <120 mmHg resulted in lower rates of cardiovascular events and cardiovascular mortality compared to a target SBP of <140 mmHg. Similarly, HYVET demonstrated that intensive treatment of hypertension in adults aged 80 years or older resulted in reduced risk of death from stroke and any cause compared to placebo.^13^ The STEP randomised controlled trial demonstrated that in adults aged 60–80 years, hypertension treatment to a target of 110–130 mmHg resulted in significantly lower incidence of stroke, heart failure and acute coronary syndrome without any increased risk of syncope or fractures.^14^ However, a criticism of HYVET and STEP has been that participants with severe multimorbidity and disability (e.g., reporting difficulties in basic activities of daily living) were excluded and therefore their results may not be applicable to frail older people.^15^

The most recent 2018 European Society of Cardiology/European Society of Hypertension (ESC/ESH) guideline for the management of arterial hypertension recommend that in older adults (i.e., aged 65 years or over), practitioners aim to an SBP target of <140 mmHg, avoiding a reading of <130 mmHg.^16^ This represents a lower SBP threshold than in the previous 2013 guidelines, where the recommended SBP target was 140–150 mmHg. The 2018 guideline also recommends that to prevent tissue hypoperfusion, diastolic BP (DBP) should be ≥70 mmHg during treatment.^17^ However, the 2018 guideline also advises to monitor frail older adults for tolerability and potential adverse effects of BP lowering, and rather non-specifically concludes that for some frail older adults, a higher BP than the recommended target may be required because the benefits of BP lowering treatment in this group has yet to be determined. In addition, there is no formal guidance as to how frailty should be identified in older adults.

Frailty can stem from different physiological dysregulations in different individuals, which may differentially influence the risks of adverse events in the context of hypertension management.^18^^,^^19^ This complexity is further compounded by the many different frailty operationalisations that have emerged in the past two decades, where each definition of frailty is based on different considerations regarding the presence of morbidities, functional impairments, disabilities, and even wider psychosocial factors.^20^^,^^21^ In this light, the aim of our study was to compare two different frailty identification schemes in their ability to identify short-term (i.e. 2-year) adverse events associated with intensive BP control (as per 2018 ESC/ESH guideline) in community-dwelling older (aged ≥ 65 years) adults being treated for hypertension who were included in the first wave of The Irish Longitudinal Study on Ageing (TILDA).

## Methods

### Study design and setting

#### Sample

TILDA is a nationally representative longitudinal cohort study of the health, economic and social circumstances of 8504 community-dwelling adults over the age of 50 in Ireland. TILDA aims to enhance the understanding of the factors that may contribute to successful ageing in this cohort and from this, inform health, social and economic policies in Ireland. TILDA's methodology and design have been described in detail by Kearney et al.^22^ TILDA utilised the Irish Geo-Directory to randomly select postal addresses and recruited participants at these addresses aged 50 years or older to formulate the Wave 1 (October 2009–June 2011) of data. The household response rate at Wave 1 was 62%.^23^ The assessments in Wave 1 included participants answering a self-completion questionnaire (SCQ), completing a computer assisted personal interview (CAPI), and undergoing a comprehensive health assessment. The Wave 1 health assessment was performed by trained research nurses in a designated TILDA Health Assessment Centre or (to a more limited extent) in the participants’ home. Wave 2 of the study (non-health assessment wave) took place approximately two years later (February 2012 – March 2013), and the participants underwent a repeat SCQ and CAPI. For the present study, data of participants in Wave 1 (baseline) and Wave 2 (follow-up) were analysed.

#### Baseline characteristics

At Wave 1, we considered the following characteristics:

• Treated for hypertension and regularly taking any of the drugs included within the following Anatomical Therapeutic Chemical (ATC) Classification System codes (https://www.whocc.no/atc_ddd_index/): C02 (antihypertensives), C03 (diuretics), C07 (beta blocking agents), C08 (calcium channel blockers) or C09 (agents acting on the renin-angiotensin system).
• Frailty by Fried's Frailty Phenotype (FP): this was defined as the presence of three or more criteria among the following: unintentional weight loss, self-reported exhaustion, weakness (by grip strength), slow walking speed and low physical activity. The operationalisation of the frailty phenotype in our study was the same as in the original Cardiovascular Health Study^24^ except for the physical activity criterion, for which we used the short form of the International Physical Activity Questionnaire.^25^^,^^26^
• Frailty by Clinical Frailty Scale (CFS): the CFS was operationalised at Wave 1 of TILDA according to the published CFS Decision Tree.^27^^,^^28^ A CFS level of 5 or more defined frail status in our analyses (non-frail if 4 or less).
• Blood pressure level. Each participant in Wave 1 had two separate blood pressure measurements recorded whilst in upright seated position, taken one minute apart, using the OMRON^TM^ digital automatic blood pressure monitor (MODEL M10-IT) with arm cuff. The average of these two readings was calculated and used as the reference blood pressure value for each participant. Intensive blood pressure treatment was defined, in keeping with the ESC/ESH 2018 guideline, as SBP <130 mmHg and/or DBP <70 mmHg.

#### Baseline groups

**Figure 1:**
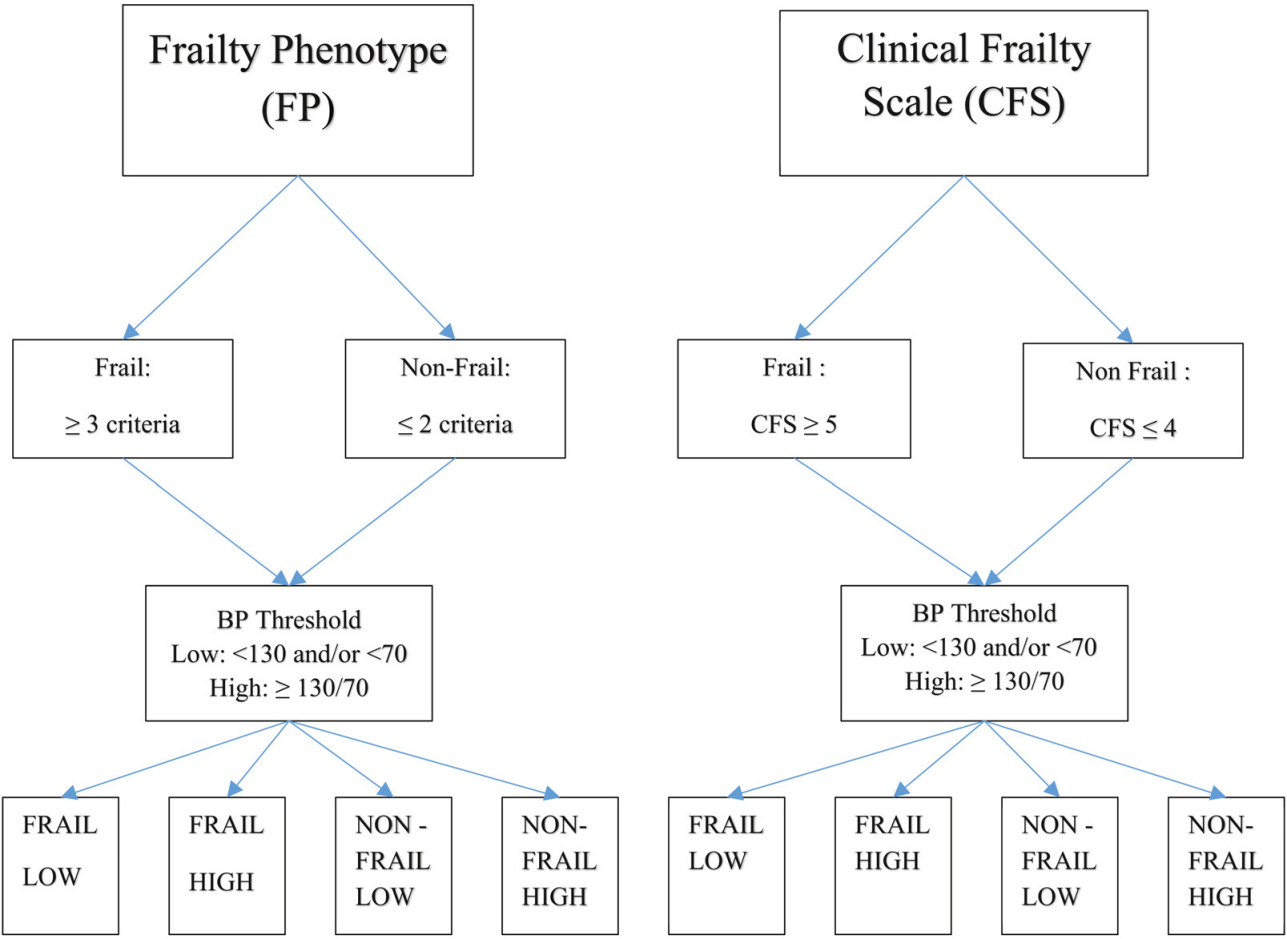
Organisation chart of frailty/blood pressure groups. Participants frail by FP if they meet 3 or more of the FP criteria. Participants frail by CFS with a score of ≥5. Low Blood Pressure (BP) defined as systolic BP < 130 mmHg and/or < 70 mmHg. High Blood Pressure (BP) defined as systolic BP ≥130/70. Abbreviations: FRAIL LOW = frail and low BP, FRAIL HIGH = frail and high BP. NON-FRAIL LOW = Non-frail and low BP, NON-FRAIL HIGH = Non-frail and high BP. FP = Frailty Phenotype, CFS= Clinical Frailty Scale.

We included Wave 1 participants aged 65 years or over, with self-reported history of hypertension who were treated with any of the above-specified medications. Based on the frailty status of a participant as determined separately by the FP and CFS and whether their blood pressure was ‘high’ or ‘low’, 8 individual BP/frailty groups were generated (Figure 1).

#### Follow-up characteristics

The following self-reported Wave 2 outcomes were analysed:

• Any new falls or fractures (hip, spine, wrist or other) since their first interview at Wave 1.
• Any new syncope, defined as having a faint or blackout since the first interview.
• Hospitalisation, stroke or TIA, heart failure and heart attack that had been newly diagnosed or occurred since their initial assessment in Wave 1.
• Mortality of Wave 1 participants was also recorded by Wave 2.

#### Statistical analysis

All statistical analyses were performed using IBM SPSS Statistics for Windows (Version 26.0. Armonk, NY: IBM Corp.). Descriptive statistics were given as mean with standard deviation (SD), median with interquartile range (IQR) or number (n) with percentage (%). To assess differences between the characteristics of each of the two sets of four blood pressure/frailty groups at baseline in Wave 1, the Chi-square test was used for dichotomous variables and the Kruskal-Wallis test for continuous variables.

To assess the longitudinal associations between each of the eight blood pressure/frailty groups and the outcomes at Wave 2, binary logistic regression models were utilised. Multicollinearity checks among predictors were conducted prior to regression analyses. In the first step, each regression was adjusted for age and sex only. In a second step, adjustment was made for age, sex, and the following additional Wave 1 covariates:

• Number of chronic medical conditions, counted from the following list: heart attack or heart failure or angina, cataracts, hypertension, high cholesterol, stroke, diabetes, lung disease, asthma, arthritis, osteoporosis, cancer, Parkinson's disease, peptic ulcer, and hip fracture.
• Orthostatic hypotension (OH), defined as a drop of ≥20 mmHg in SBP and/or ≥10 mmHg DBP on standing from a seated position, which was also measured in TILDA as described elsewhere.^29^
• Polypharmacy, defined as a participant being on 5 or more regular medications.
• Low level of education, defined as educated up to primary school level.
• Montreal Cognitive Assessment (MOCA) score.^30^

Forest plots were created with Microsoft Excel to represent the fully adjusted Odds Ratio (OR) for each of the frailty/BP groups and outcomes studied, noting the 95% Confidence Interval (CI) for each OR and associated P value. Statistical significance was set at *P* < 0.05.

The study reporting was done in compliance with STROBE guidelines.^31^

#### Ethical considerations

Each TILDA wave received approval by the Faculty of Health Sciences Research Ethics Committee at Trinity College Dublin. All participants provided written informed consent prior to inclusion in the study. All experimental procedures adhered to the Declaration of Helsinki.

#### Role of the funding source

The Irish Longitudinal Study on Ageing is funded by the Irish Government Department of Health, the Atlantic Philanthropies and Irish Life plc. Roman Romero-Ortuno is funded by a Grant from Science Foundation Ireland under Grant number 18/FRL/6188. The financial sponsors played no role in the design, execution, analysis, and interpretation of data, or write up of the study.

All authors had full access to the TILDA database. PO'D, AO'H, RAK and RRO made the decision to submit the manuscript for publication.

## Results

### Baseline characteristics of participants

**Figure 2:**
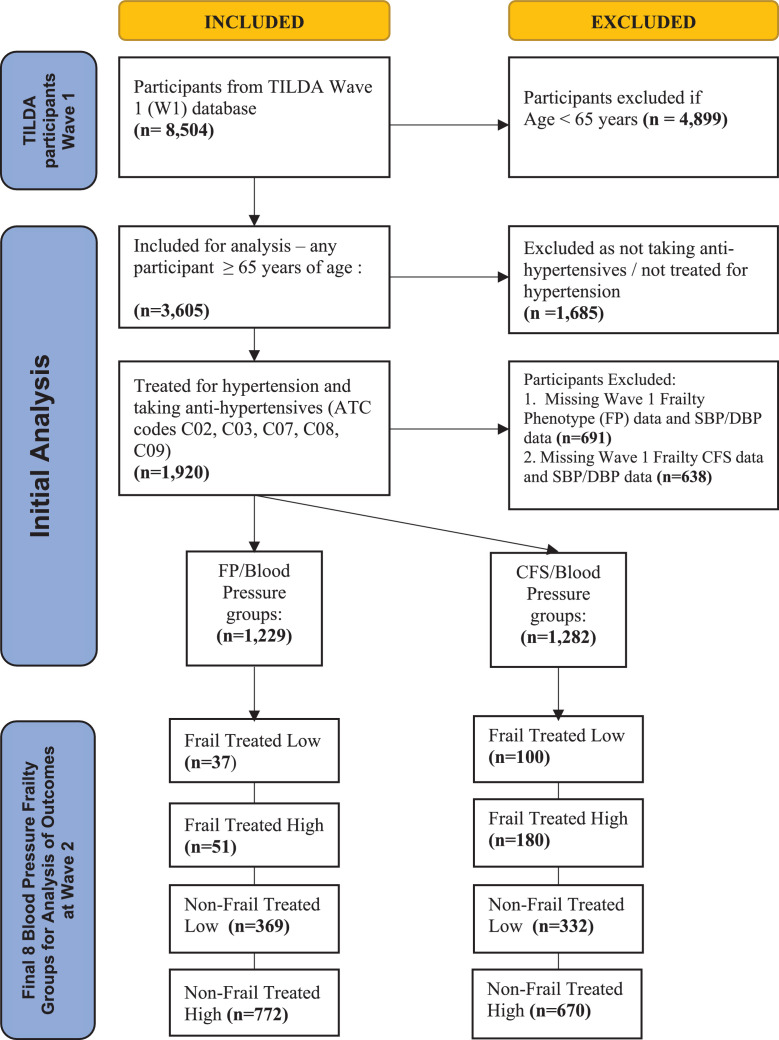
Flow diagram of selection of participants for analysis. Participants from W1 of TILDA included in final analysis: aged ≥ 65 years of age and being treated with medication for hypertension. Subsequently divided into frailty/blood pressure groups based on W1 frailty status and BP reading. Abbreviations: FP = frailty phenotype, CFS = clinical frailty scale, ATC code = anatomical therapeutic chemical code, BP=blood pressure, W1= Wave 1.

Of the original 8504 Wave 1 participants, 3605 were aged 65 years or more and 1920 were on medication to treat hypertension. Of the 1920, 108 participants were deceased by Wave 2 and a further 223 participants were lost to follow up. 1229 had complete data for both FP and BP, and 1282 for CFS and BP as outlined in Figure 2. None of the TILDA Wave 1 participants were classified in the CFS 8 (very severely frail) or 9 (terminally ill) categories, as noted elsewhere.^27^

In the FP total sample of 1229, 88 (7.1%) were frail and in the CFS sample of 1282, 280 (21.8%) were frail. Among the latter, 59 (21.1%) were mildly frail, 183 (65.4%) had moderate frailty and 38 (13.6%) were severely frail. The Chi-square association between the two dichotomous frailty measures was significant (*P* < 0.001).

The baseline characteristics at Wave 1 and the proportions of Wave 2 outcomes of the 8 different blood pressure/frailty groups are outlined in Tables 1 (FP) and 2 (CFS). Participants who were in the groups that were frail (by FP or CFS) with low or high blood pressure seemed older (FP: *P* < 0.001, CFS: *P* < 0.001), have a higher number of chronic diseases (FP: *P* < 0.001, CFS: *P* < 0.001) and more polypharmacy (FP: *P* < 0.001, CFS: *P* < 0.001) than the non-frail blood pressure groups.

**Table 1:** Baseline characteristics and outcomes of bp-frailty groups – frailty by phenotype, BP threshold SBP < 130 and/or DBP < 70 mmHg.

	Frail Treated Low*n* = 37	Frail Treated High*n* = 51	Non-Frail Treated Low*n* = 369	Non-Frail Treated High*n* = 772	*P* Value (overall difference)
**Wave 1 characteristics**					
Mean Age (SD)	76.0 (6.9)	79.3 (6.3)	73.2 (5.8)	73.3 (6.4)	<0.001*
Female Sex (%)	13 (35.1)	24 (47.1)	190 (51.5)	371 (48.1)	0.255#
Education level: up to primary only (%)	17 (45.9)	34 (66.7)	160 (43.4)	314 (40.8)	0.004#
Median number of chronic diseases (IQR)	3 (3)	3 (3)	3 (2)	3 (2)	<0.001*
Median number of physical limitations (IQR)	6 (4)	6 (4)	2 (3)	2 (3)	<0.001*
Median MOCA score (IQR)	20 (7)	21 (7)	24 (6)	24 (6)	<0.001*
Polypharmacy (%)	32 (86.5)	37 (72.5)	213 (57.7)	359 (46.5)	<0.001#
Mean seated SBP mmHg (SD)	119.2(13.0)	158.0(19.03)	121.7(11.5)	151.5 (16.8)	<0.001#
Mean seated DBP mmHg (SD)	69.3(13.3)	90.6(20.1)	71.2(10.0)	87.4 (14.8)	<0.001#
Orthostatic hypotension (%)	7 (18.9)	9 (17.6)	23 (6.3)	112 (14.6)	<0.001#
**Wave 2 outcomes**					
Any fall or fracture (%)	15 (48.4)	15 (39.5)	82 (25)	207 (19.3)	0.016#
New syncope (%)	1 (4.3)	2 (8.3)	10 (3.8)	30 (5.2)	0.694#
New hospitalisation (%)	19 (61.3)	14 (36.8)	74 (22.5)	159 (22.5)	<0.001#
New stroke or TIA (%)	1 (3.2)	3 (7.9)	5 (1.5)	27 (3.8)	0.092#
New heart failure (%)	2 (6.5)	1 (2.6)	4 (1.2)	6 (0.8)	0.033#
New heart attack (%)	1 (3.8)	0 (0)	3 (1.0)	7 (1.0)	0.520#
Mortality (%)	3 (8.1)	6 (11.8)	14 (3.8)	22 (2.8)	0.005#

⁎ Kruskal-Wallis test; # Chi-Square test.

**Table 2:** Baseline characteristics and outcomes of bp-frailty groups – frailty by CFS, BP threshold SBP < 130 and/or DBP < 70 mmHg.

	Frail Treated Low*n* = 100	Frail Treated High*n* = 180	Non-Frail Treated Low*n* = 332	Non-Frail Treated High*n* = 670	*P* Value (overall difference)
**Wave 1 characteristics**					
Mean Age (SD)	76.1 (6.7)	76.9 (7.2)	73.1 (5.7)	73.1 (6.2)	<0.001*
Female Sex (%)	57 (57.0)	95 (52.2)	161 (48.5)	317 (47.3)	0.251#
Education level: up to primary only (%)	44 (44.0)	96 (53.3)	146 (44.0)	265 (39.7)	<0.001#
Median number of chronic diseases (IQR)	4 (2)	3 (2)	3 (2)	3 (1)	<0.001*
Median number of physical limitations (IQR)	5 (3)	6 (4)	2 (3)	2 (2)	<0.001*
Median MOCA score (IQR)	22 (7)	22 (7)	23 (6)	25 (5)	<0.001*
Polypharmacy (%)	81 (81.0)	119 (66.1)	181 (54.5)	295 (44.0)	<0.001#
Mean seated SBP mmHg (SD)	120.7(16.7)	151.2(16.7)	121.7(11.4)	152.0(17.0)	<0.001*
Mean seated DBP mmHg (SD)	70.4(10.0)	88.5(19.0)	71.1(10.3)	87.3(13.8)	<0.001*
Orthostatic hypotension (%)	81 (81.0)	119 (66.1)	181 (54.5)	295 (44.0)	<0.001#
**Wave 2 outcomes**					
New fall or fracture (%)	22 (25.6)	39 (25.0)	46 (15.8)	82 (13.5)	0.001#
New syncope (%)	6 (8.8)	7 (6.3)	6 (2.6)	25 (5.0)	0.141#
New hospitalisation (%)	31 (36.0)	50 (32.1)	67 (22.9)	127 (20.9)	0.001#
New stroke or TIA (%)	2 (2.3)	10 (6.4)	6 (2.0)	20 (3.3)	0.095#
New heart failure (%)	0 (0.0)	4 (2.6)	6 (2.0)	3 (0.5)	0.042#
New heart attack (%)	2 (2.6)	1 (0.7)	3 (1.1)	6 (1.0)	0.606#
Mortality (%)	10 (10.1)	15 (8.3)	13 (3.9)	21 (3.1)	0.001#

⁎ Kruskal-Wallis test; # Chi-Square test.

### Antihypertensive medication classes

As regards the most common antihypertensive classes among the 88 participants who were frail by FP, 57 (64.8%) were taking agents acting on the renin-angiotensin system, 38 (43.2%) diuretics, 34 (38.6%) beta blockers, 21 (23.9%) calcium channel blockers, and 8 (9.1%) anti-adrenergic agents. Among the 280 who were frail by CFS, 177 (63.2%) were taking agents acting on the renin-angiotensin system, 119 (42.5%) beta blockers, 94 (33.6%) diuretics, 71 (25.4%) calcium blockers, and 25 (8.9%) anti-adrenergic agents. Polypharmacy was present in 69 (78.4%) of the frail by FP, and in 200 (71.4%) of the frail by CFS.

Of note, due to the statistical multicollinearity between the CFS and the variable ‘number of physical impairments’, the latter was not included in the full binary logistic regression models. This was also applied to the FP models, given that the FP is a pre-disability state.^32^

### Longitudinal health outcomes at wave 2

**Figure 3:**
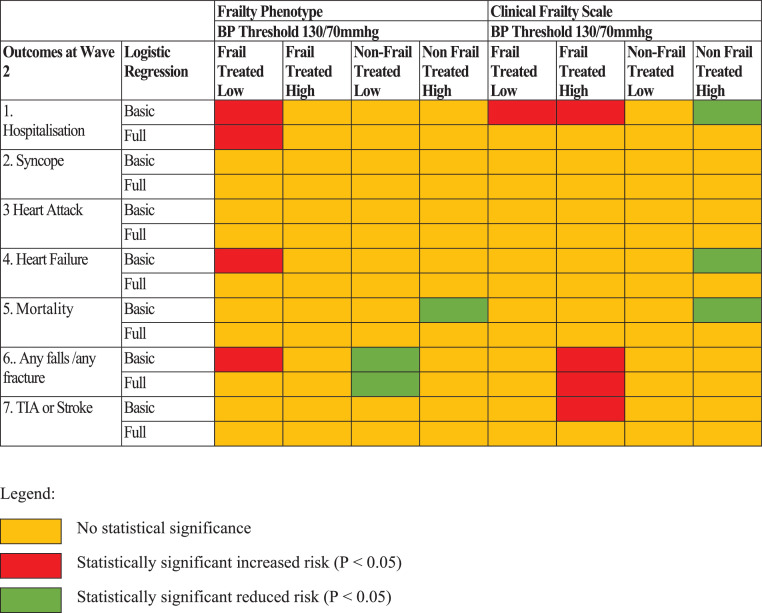
Summary of the independent effects of each frailty/blood pressure group on longitudinal outcomes after basic and full logistic regression adjustment. Basic binary logistic regression models adjusted for age and sex. Full binary logistic regression models adjusted for age, sex, education, polypharmacy, classic orthostatic hypotension (OH), Montreal Cognitive Assessment (MOCA) score and number of chronic diseases.

Figure 3 summarises the longitudinal outcomes for the 8 individual blood pressure-frailty groups at Wave 2 after basic and full logistic regression adjustment. The full regression models can be seen in Appendices 1 & 2. In the fully adjusted model, the non-frail by FP treated low had a reduced risk of falls/fractures (*P* = 0.031). For the frail groups, the frail by FP treated intensely had a statistically significant increased risk of hospitalisation by Wave 2 (*P* = 0.001). Conversely, none of the frail by CFS groups treated intensely had increased risk of any outcomes at Wave 2. The frail by CFS treated high had a significantly increased risk of falls/fractures in the fully adjusted model (*P* = 0.035), but not for any other outcomes.

**Figure 4:**
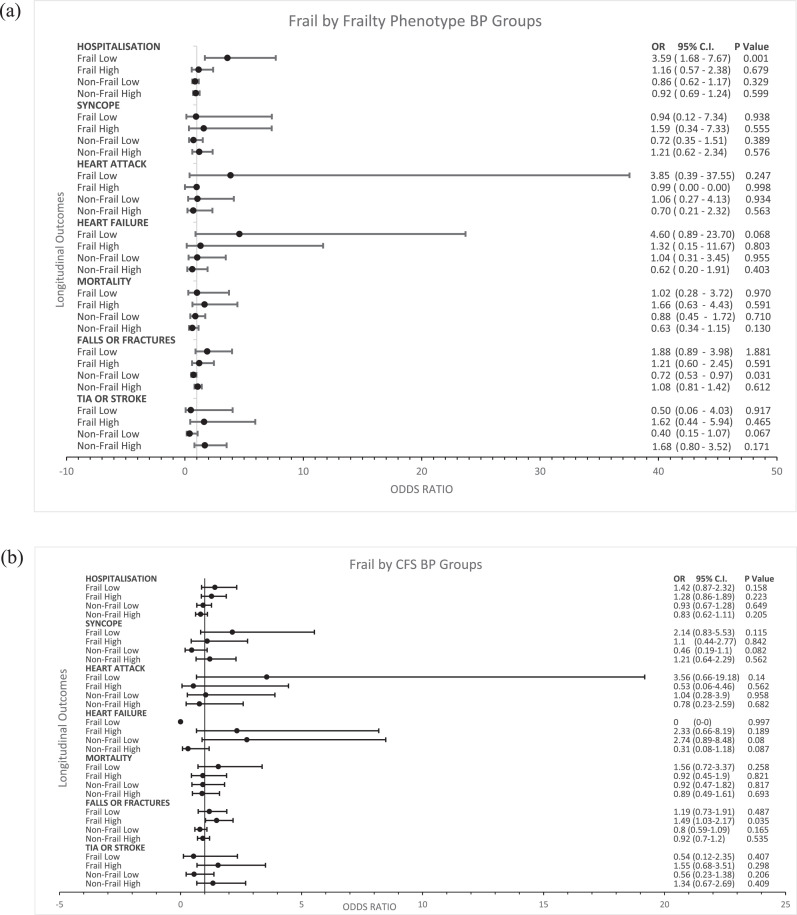
(a)Forest plot of odds ratios for Wave 2 adverse health outcomes for Frailty by Frailty Phenotype-BP Groups from Wave 1 This figure shows the statistically significant association between the frail by FP treated low group and increased risk of hospitalisation at Wave 2. It also shows the association between the non-frail by FP treated low group and reduced risk of falls or fractures. Error bars represent 95% confidence intervals. Odds ratios for each health outcome were plotted on the X-axis after adjustment for age, sex, education, polypharmacy, classic orthostatic hypotension (OH), Montreal Cognitive Assessment (MOCA) score and number of chronic diseases. Statistical significance was met with a *P* value of <0.05. Abbreviations: CI= confidence intervals, OR= odds ratio, TIA= transient ischaemic attack. (b) Forest plot of odds ratios for Wave 2 adverse health outcomes for Frailty by Clinical Frailty Scale (CFS)-BP Groups from Wave 1 This figure shows the statistically significant association between the frail by CFS treated high group and an increased risk of falls or fractures at Wave 2. Error bars represent 95% confidence intervals. Odds ratios for each health outcome were plotted on the X-axis after adjustment for age, sex, education, polypharmacy, classic orthostatic hypotension (OH), Montreal Cognitive Assessment (MOCA) score and number of chronic diseases. Statistical significance was met with a *P* value of <0.05. Abbreviations: CI= confidence intervals, OR= Odds Ratio, TIA= Transient Ischaemic Attack.

Figure. 4(a) (FP) and (b) (CFS) show the forest plots of the fully adjusted ORs for each of the frailty/BP groups and outcomes studied, noting the 95% CIs for the ORs and their associated P values.

## Discussion

In this study, we compared two frailty classifications in their ability to predict 2-year adverse outcomes associated with intensive BP control (<130/70 mmHg as per ESC/ESH guidelines) in TILDA. We hypothesised that hypertensive frail older adults treated intensively may experience more adverse outcomes at follow-up than those treated to higher blood pressures, and that associations with outcomes may vary according to different frailty classifications.

We found that while the FP only identified risks associated with intensive BP treatment (namely hospitalisation in the fully adjusted model), intensively treated frail-by-CFS participants had no increased or decreased risk of adverse outcomes in the fully adjusted models, but those treated above the target had a higher risk of falls/fractures. Interestingly, in the non-frail by FP, intensive blood pressure treatment was associated with a reduced risk of falls/fractures.

Our results indicate that the interaction between frailty and blood pressure control is more nuanced and complex than current guidelines may suggest. In our study, frailty status was the predominant discriminating factor in predicting the outcomes at Wave 2 for the 8 blood-pressure frailty groups. Of the 4 groups where the participants were not frail by FP or CFS, with high or low BP, there was no association with any of the adverse outcomes reviewed at Wave 2. Any significantly higher risk of adverse health outcomes in Wave 2 only occurred in frail groups. This finding reflects previous studies where frailty has been shown to increase the risk of adverse health outcomes such as falls, fracture, delirium, hospitalisation, physical limitations, and mortality.^9^^,^^33^

Specific reasons for hospitalisation were not available, but given that in the basic models, frail-by-FP participants treated to a low BP had a statistically significant risk of falls/fractures and incident heart failure, hospitalisations may be hypothetically related to those outcomes. Frailty by phenotype is a pre-disability state and incorporates physical attributes such as muscle weakness, slow gait speed and low physical activity, which have been shown to be indicative of low musculoskeletal mass, or sarcopaenia.^34^ These three aspects of the FP in conjunction with sarcopenia may contribute to decline in the ‘muscle pump’, potentially increasing the risk of orthostatic hypotension, and in turn conferring higher risk of reduced balance, falls^35^^,^^36^ and potentially fractures. This could be related to the risk of hospitalisation seen at Wave 2, potentially from falls,^37^ orthostatic hypotension,^38^ and/or syncope.^39^ Furthermore, the typical clinical aspects of heart failure, especially in its advanced stages, have been reported to overlap considerably with the manifestations of physical frailty – self-reported exhaustion and low physical activity in particular^40^; indeed, in advancing heart failure, patients may have low blood pressure (‘cardiac pump’ failure) and still require cardiovascular medications. Alternatively, the physiological dysregulation of cardiovascular homeostasis seen in the frailty state combined with the fact that low BP is common in heart failure with reduced ejection fraction,^41^ and associated with hospitalisation for heart failure,^42^ could potentially result in the frail by FP treated low having a possible prospective association with heart failure.

On the other hand, frailty by CFS includes chronic conditions^43^ and disability in its definition to a greater extent than the FP.^28^ The fact that frail-by-CFS treated high had a higher risk of future falls may mean that those falls are less related to orthostatic hypotension than potentially to gait and balance disorders and/or cognitive decline, together with specific morbidities and/or disabilities. Therefore, independent of BP status, the frailer by CFS a person is the more comorbidities and functional decline endured and thus the greater potential need for hospital care. The fact that frail-by-CFS participants treated to a high BP had an increased risk of falls/fractures in the full model could also be hypothetically related to the fact that in these more visibly disabled participants, their medical practitioners may have already deprescribed antihypertensives to avoid orthostatic hypotension/falls, but most of the falls could in fact be driven by poor balance/gait disorders related to the disability itself.

Limitations of our study include that TILDA is a population-based study of community-dwelling adults and has a relatively low number of frail participants. In addition, from Wave 1 to Wave 2, there was some attrition with 223 participants lost to follow up. The numbers of participants in the frailty groups were small because even though TILDA recruited participants aged 50 years and over, frailty is a geriatric syndrome and as such it is generally accepted that it is applicable to those aged 65 and over. Indeed, the FP and the CFS were originally validated in the 65+ population.^24^^,^^44^ Furthermore, for the purposes of the ESC/ESH hypertension guidelines, ‘older’ patients were defined as those aged ≥65 years. For these reasons, we excluded the participants in TILDA aged between 50 and 64 and this drove our numbers down. This may have resulted in reduced statistical power, suggested by the wide CIs for some groups in the forest plots, and loss of statistical significance after full adjustment. Therefore, the major limitation of this study is that there are likely to be insufficient numbers of people within frailty strata other than non-frail to provide sufficient power, and no power calculation a priori was conducted because this was a secondary analysis of an existing dataset.

Consequently, our results may not be generalisable to clinical populations seen for instance in the acute hospital. Our study had only 38 participants who were classified as ‘severely frail’ with a CFS of 7 and did not include participants with more advanced frailty level (i.e. CFS 8 or 9). Therefore, most of the frail participants in this study are arguably those with mild to moderate frailty and not necessarily representative of those with very advanced levels of dependency and limited life expectancy, where outcomes with intensive blood pressure control could be potentially very different. There is a well-described terminal decline in SBP in the last 24 months of life^10^ and this patient group is not represented in the TILDA cohort. A further limitation of our study is that FP and CFS classifications may overlap (however only 57 participants were classified as frail by both). Independent analyses on two non-overlapping frailty classifications may have helped sharpen differences in clinical outcomes. However, only 33 participants were frail by FP but not by CFS and therefore this analysis would be severely underpowered. Similarly, numbers were insufficient to statistically analyse the 57 participants in the FP/CFS overlap group, especially when the numbers reduce further once they are sub-divided into ‘low’ or ‘high’ BP groups. A significant proportion of the frail participants had polypharmacy and the most common antihypertensive drug classes taken were drugs acting on renin-angiotensin system, beta blockers and diuretics. Similarly, we did not have the power within the frailty/BP subgroups to study individual drug classes and their relationship with any of the outcomes. This is as important aspect to be assessed in future studies.1.

In other studies such as the HYVET, SPRINT and STEP trials, participants were not categorised by frailty status in their original studies. Pajewski et al. constructed a 36-item frailty index (FI) in the SPRINT participants and reported that their frail participants had double the rate of falls, and three times the rate of all-cause hospitalisations compared to fit participants but did not assess mortality.^45^ Importantly, the interaction between frailty status and BP treatment was not assessed. A similar analysis done on the HYVET participants assessed the connection between treatment effect of hypertension and frailty via a FI, finding no interaction between baseline FI and BP treatment for risk of all-cause mortality, stroke or cardiovascular events.^46^ An innovation of our study compared to SPRINT is that we specifically assessed the interaction between frailty and BP status on multiple health outcomes including mortality. Similarly to HYVET, our study assessed hospitalisations, falls/fractures and syncope, as well as mortality and cardiovascular outcomes.

An important point to note in our study is that we assessed both frailty and BP status as being dichotomous. Without doubt, categorising frailty as ordinal and BP as continuous would have provided more information in the analysis. However, we thought that dichotomising both frailty and BP would be clearer from the point of view of the guideline recommendations. Indeed, the ESC/ESH guideline refers to a patient being “frail” and does not discuss “degrees of frailty”.^16^ Similarly, the guideline also gives absolute targets for BP being high or low. In consequence, we designed our research with the aim of being consistent with the dichotomous classifications provided by the guideline.

In conclusion, our results illustrate how different frailty tools capture different health outcomes when applying a hypertension management guideline. While it is very welcome that the latest ESC/ESH hypertension guideline considers frailty, frailty is too heterogenous to fit into a one-size fits all approach. For the non-frail older adults, the guidelines advocate that BP should be lowered to <140/80 mmHg if tolerated but not below 130 mmHg. For frail older people, guidelines should be more specific as to what frailty model is operationalised to identify those at risk of health outcomes from intensively treated BP. This has been highlighted in a recent systematic review by Bogaerts et al. who concluded that current hypertension treatment recommendations in older adults are inconsistent and based on chronological age rather than a patient's frailty status or biological age.^47^ From our study, the frail did not necessarily suffer more adverse outcomes from intensely treated BP but rather it varied depending on the frailty model utilised and the specific outcome assessed. Therefore, even when utilising frailty tools for identification of at-risk patients, a full geriatric assessment and personalisation of treatment plans remains necessary. We feel that our contribution highlights some gaps in the evidence that should be addressed by future research in clinical studies. Our population-based, observational study aims to generate awareness of the need for further similar studies with larger power and in more clinical populations to identify the real world clinical implications of hypertension treatment in frail older adults, where the properties of different frailty classification schemes can be studied and compared. These independent, higher-powered studies with a subsequent meta-analysis would address this current gap in the evidence and help definitively inform future clinical guidelines.

## Declaration of interests

We declare no competing interests.

## Supplementary materials

Image, application 1

Image, application 2

Image, application 2

Image, application 2
