# Supplementary material for: Do the frail experience more adverse events from intensive blood pressure control? A 2-year prospective study in the Irish Longitudinal Study on Ageing (TILDA)
Source: eClinicalMedicine. 2022 Feb 19;45:101304. doi: 10.1016/j.eclinm.2022.101304 (PMC8860911; doi:10.1016/j.eclinm.2022.101304)
Supplement: Supplementary file 1 [file mmc1.docx]

**Appendix 1. Binary Logistic Regression Analysis for outcomes at Wave 2 for 4 Groups with Frailty by Frailty Phenotype (FP)**

**Index :**

Outcome 1 Hospitalisation: Page 2-5

Outcome 2 Syncope: Page 6-9

Outcome 3 Heart Attack: Page 10-13

Outcome 4 Heart Failure: Page 14-17

Outcome 5 Deceased by Waave 2: Page 18-21

Ouctome 6 Falls or Fractures: Page 22-25

Outcome 7 TIA or Stroke: Page 26-29

**Abbreviations/Legend:**

FPlow **–** Frail by phenotype with blood pressure treated low

FPhigh – Frail by phenotype with blood pressure treated high

NonFPlow – Non-frail by phenotype with blood pressure treated low

NonFPhigh – Non-frail by phenotype with blood pressure treated high

CHRchronic **-** Number of chronic medical conditions, counted from the following list: heart attack or heart failure or angina, cataracts, hypertension, high cholesterol, stroke, diabetes, lung disease, asthma, arthritis,

MDpolypharmacy **-** Polypharmacy, defined as a participant being on 5 or more regular medications.

MOCASum - Montreal Cognitive Assessment (MOCA) score

LOWEDU - Low level of education, defined as educated up to primary school level.

CLASSICOH **-** defined as a drop of ≥20 mmHg in SBP and/or ≥10 mmHg DBP on standing from a seated position, which was also measured in TILDA as described elsewhere

**Outcome 1. Hospitalisation by Wave 2**

**(i) Frail by phenotype treated low – Basic Logistic Regression Model**

|  | | **Odds Ratio** | **95% C.I. 95% C.I.** | |  |
| --- | --- | --- | --- | --- | --- |
|  |  |  | **Lower** | **Upper** | **P Value** |
|  | FPlow | 4.868 | 2.312 | 10.248 | 0.000 |
|  | age | 1.032 | 1.010 | .000 | 0.005 |
|  | sex | .663 | .501 | .005 | 0.004 |
|  | Constant | .055 |  |  | 0.000 |

**(ii) Frail by phenotype treated low – Full Binary Logistic Regression Model**

|  | | **Odds Ratio** | | **95% C.I. 95% C.I.** | |  |
| --- | --- | --- | --- | --- | --- | --- |
|  |  |  |  | **Lower** | **Upper** | **P Value** |
|  | FPlow | | 3.592 | 1.682 | 7.674 | 0.001 |
|  | age | | 1.023 | .999 | .001 | 0.055 |
|  | sex | | .610 | .454 | .055 | 0.001 |
|  | CHRchronic | | 1.111 | 1.007 | .001 | 0.036 |
|  | MDpolypharmacy | | 1.662 | 1.231 | .036 | 0.001 |
|  | MOCASum | | .969 | .932 | .001 | 0.102 |
|  | LOWEDU | | .991 | .729 | .102 | 0.954 |
|  | CLASSICOH | | 1.125 | .731 | .954 | 0.591 |
|  | Constant | | .132 |  | .591 | 0.077 |

**(iii) Frail by Phenotype treated high – Basic Logistic Regression Model**

|  | | **Odds Ratio** | **95% C.I. 95% C.I.** | | |  | |
| --- | --- | --- | --- | --- | --- | --- | --- |
|  |  |  | **Lower** | | **Upper** | **P Value** | |
|  | FPhigh | 1.502 | | .751 | 3.006 | | 0.250 |
|  | age | 1.036 | | 1.014 | 1.059 | | 0.001 |
|  | sex | .630 | | .477 | .832 | | 0.001 |
|  | Constant | .044 | |  |  | | 0.000 |

**(iv) Frail by phenotype treated high – Full Binary Logistic Regression Model**

|  | | **95% C.I.** | | **95% C.I.** | |
| --- | --- | --- | --- | --- | --- |
|  |  | **Odds Ratio** | **Lower** | **Upper P Value** | |
|  | FPhigh | 1.163 | .568 | 2.380 | .679 |
|  | age | 1.026 | 1.002 | 1.050 | .037 |
|  | sex | .565 | .421 | .759 | .000 |
|  | CHRchronic | 1.131 | 1.026 | 1.247 | .014 |
|  | MDpolypharmacy | 1.671 | 1.241 | 2.250 | .001 |
|  | MOCASum | .953 | .917 | .990 | .014 |
|  | LOWEDU | .972 | .715 | 1.322 | .859 |
|  | CLASSICOH | 1.246 | .815 | 1.905 | .310 |
|  | Constant | .180 |  |  | .132 |

**(v) Non-frail by phenotype treated low – Basic Logistic Regression Mode**

|  | | **95% C.I 95% C.I.** | | |  |
| --- | --- | --- | --- | --- | --- |
|  |  | **Odds Ratio** | **Lower Upper** | | **P Value** |
|  | NonFPlow | .913 | .671 | 1.241 | .560 |
|  | age | 1.035 | 1.013 | 1.058 | .002 |
|  | sex | .647 | .489 | .855 | .002 |
|  | Constant | .047 |  |  | .000 |

**(vi) Non-frail by phenotype treated low –Full Binary Logistic Regression Model**

|  | | **Odds Ratio** | **95% C.I. 95% C.I.** | |  |
| --- | --- | --- | --- | --- | --- |
|  |  |  | **Lower** | **Upper** | **P Value** |
|  | NonFPlow | .855 | .623 | 1.171 | .329 |
|  | age | 1.023 | .999 | .329 | .060 |
|  | sex | .595 | .444 | .060 | .001 |
|  | CHRchronic | 1.127 | 1.022 | .001 | .016 |
|  | MDpolypharmacy | 1.743 | 1.292 | .016 | .000 |
|  | MOCASum | .962 | .926 | .000 | .044 |
|  | LOWEDU | .973 | .717 | .044 | .861 |
|  | CLASSICOH | 1.151 | .751 | .861 | .518 |
|  | Constant | .172 |  | .518 | .122 |

**(vii) Non-frail by phenotype treated high – Basic Logistic Regression Model**

|  |  | |  |
| --- | --- | --- | --- |
|  | **Odds Ratio** | **95% C.I. Lower 95% C.I. Upper** | **P Value** |

|  | | **Odds Ratio** | **95% C.I. 95% C.I.** | | **P Value** |
| --- | --- | --- | --- | --- | --- |
|  |  |  | **Lower** | **Upper** |  |
|  | NonFPhigh | .800 | .121 | 1.061 | .121 |
|  | age | 1.039 | .000 | 1.062 | 0.000 |
|  | sex | .619 | .001 | .820 | 0.001 |
|  | Constant | .043 | .000 |  | 0.000 |

**(viii) Non-frail by phenotype treated high – Full Binary Logistic Regression Model**

|  | | **Odds Ratio** | **95% C.I. 95% C.I.** | |  |
| --- | --- | --- | --- | --- | --- |
|  |  |  | **Lower** | **Upper** | **P Value** |
|  | NonFPhigh | .924 | .690 | 1.239 | .599 |
|  | age | 1.026 | 1.002 | .599 | .031 |
|  | sex | .564 | .421 | .031 | .000 |
|  | CHRchronic | 1.131 | 1.026 | .000 | .013 |
|  | MDpolypharmacy | 1.659 | 1.230 | .013 | .001 |
|  | MOCASum | .954 | .918 | .001 | .015 |
|  | LOWEDU | .977 | .719 | .015 | .880 |
|  | CLASSICOH | 1.263 | .826 | .880 | .282 |
|  | Constant | .180 |  | .282 | .131 |

**Outcome 2. Syncope by Wave 2**

**(i) Frail by phenotype treated low – Basic Logistic Regression Model**

|  | | **Odds Ratio** | **95% C.I. 95% C.I.** | |  |
| --- | --- | --- | --- | --- | --- |
|  |  |  | **Lower** | **Upper** | **P Value** |
|  | FPlow | .893 | .117 | 6.837 | .913 |
|  | age | 1.018 | .971 | 1.067 | .463 |
|  | sex | 1.292 | .697 | 2.393 | .416 |
|  | Constant | .009 |  |  | .010 |

**(ii) Frail by phenotype treated low – Full Binary Logistic Regression Model**

|  | | **Odds Ratio** | **95% C.I. 95% C.I.** | |  |
| --- | --- | --- | --- | --- | --- |
|  |  |  | **Lower** | **Upper** | **P Value** |
|  | FPlow | .921 | .116 | 7.336 | .938 |
|  | age | 1.028 | .976 | 1.083 | .293 |
|  | sex | 1.440 | .754 | 2.749 | .269 |
|  | CHRchronic | .915 | .729 | 1.148 | .444 |
|  | MDpolypharmacy | 1.710 | .873 | 3.349 | .118 |
|  | MOCASum | 1.076 | .986 | 1.175 | .102 |
|  | LOWEDU | 1.600 | .827 | 3.096 | .163 |
|  | CLASSICOH | 1.456 | .633 | 3.349 | .377 |
|  | Constant | .000 |  |  | .003 |

**(iii) Frail by phenotype treated high – Basic Logistic Regression Model**

|  | | **Odds Ratio** | **95% C.I. 95% C.I.** | |  |
| --- | --- | --- | --- | --- | --- |
|  |  |  | **Lower** | **Upper** | **P Value** |
|  | FPhigh | 1.588 | .351 | 7.198 | .548 |
|  | age | 1.025 | .978 | 1.074 | .309 |
|  | sex | 1.339 | .726 | 2.467 | .350 |
|  | Constant | .005 |  |  | .004 |

**(iv) Frail by phenotype treated high – Full Binary Logistic Regression Model**

|  | | **Odds Ratio** | **95% C.I. 95% C.I.** | |  |
| --- | --- | --- | --- | --- | --- |
|  |  |  | **Lower** | **Upper** | **P Value** |
|  | FPhigh | 1.586 | .343 | 7.325 | .555 |
|  | age | 1.034 | .983 | 1.088 | .198 |
|  | sex | 1.482 | .780 | 2.812 | .229 |
|  | CHRchronic | .916 | .732 | 1.146 | .442 |
|  | MDpolypharmacy | 1.551 | .803 | 2.996 | .191 |
|  | MOCASum | 1.066 | .979 | 1.161 | .142 |
|  | LOWEDU | 1.606 | .835 | 3.089 | .156 |
|  | CLASSICOH | 1.468 | .642 | 3.359 | .363 |
|  | Constant | .000 |  |  | .002 |

**(v) Non -frail by phenotype treated low – Basic Logistic Regression Model**

|  | | **Odds Ratio** | **95% C.I. 95% C.I.** | |  |
| --- | --- | --- | --- | --- | --- |
|  |  |  | **Lower** | **Upper** | **P Value** |
|  | NonFPlow | .714 | .346 | 1.475 | .363 |
|  | age | 1.017 | .970 | 1.066 | .493 |
|  | sex | 1.312 | .708 | 2.432 | .389 |
|  | Constant | .011 |  |  | .013 |

**(vi) Non-frail by phenotype treated low – Full Binary Logistic Regression Model**

|  | | **Odds Ratio** | **95% C.I. 95% C.I.** | | **P Value** |
| --- | --- | --- | --- | --- | --- |
|  |  |  | **Lower** | **Upper** |  |
|  | NonFPlow | .723 | .345 | 1.514 | .389 |
|  | age | 1.026 | .975 | 1.081 | .325 |
|  | sex | 1.468 | .768 | 2.804 | .245 |
|  | CHRchronic | .912 | .727 | 1.144 | .426 |
|  | MDpolypharmacy | 1.763 | .898 | 3.463 | .100 |
|  | MOCASum | 1.076 | .986 | 1.175 | .099 |
|  | LOWEDU | 1.602 | .828 | 3.102 | .162 |
|  | CLASSICOH | 1.384 | .599 | 3.197 | .447 |
|  | Constant | .001 |  |  | .003 |

**(vii) Non-frail by phenotype treated high – Basic Logistic Regression Model**

|  | | **Odds Ratio** | **95% C.I. 95% C.I.** | |  |
| --- | --- | --- | --- | --- | --- |
|  |  |  | **Lower** | **Upper** | **P Value** |
|  | NonFPhigh | 1.261 | .658 | 2.418 | .485 |
|  | age | 1.028 | .982 | 1.077 | .239 |
|  | sex | 1.335 | .724 | 2.459 | .355 |
|  | Constant | .004 |  |  | .002 |

**(viii) Non-frail by phenotype treated low – Full Binary Logistic Regression Model**

|  | | **Odds Ratio** | **95% C.I. 95% C.I.** | |  |
| --- | --- | --- | --- | --- | --- |
|  |  |  | **Lower** | **Upper** | **P Value** |
|  | NonFPhigh | 1.210 | .621 | 2.356 | .576 |
|  | age | 1.035 | .985 | 1.089 | .175 |
|  | sex | 1.476 | .778 | 2.800 | .233 |
|  | CHRchronic | .921 | .736 | 1.152 | .471 |
|  | MDpolypharmacy | 1.592 | .821 | 3.089 | .169 |
|  | MOCASum | 1.062 | .975 | 1.157 | .165 |
|  | LOWEDU | 1.626 | .845 | 3.127 | .145 |
|  | CLASSICOH | 1.441 | .628 | 3.306 | .389 |
|  | Constant | .000 |  |  | .001 |

**Outcome 3 : Heart Attack by Wave 2**

**(i) Frail by phenotype treated low – Basic Logistic Regression Model**

|  | | **Odds Ratio** | **95% C.I. 95% C.I.** | |  |
| --- | --- | --- | --- | --- | --- |
|  |  |  | **Lower** | **Upper** | **P Value** |
|  | FPlow | 4.190 | .501 | 35.063 | .186 |
|  | age | .968 | .874 | .186 | .539 |
|  | sex | .666 | .192 | .539 | .521 |
|  | Constant | .180 |  | .521 | .653 |

**(ii) Frail by phenotype treated low – Full Binary Logistic Regression Model**

|  | | **Odds Ratio** | **95% C.I. 95% C.I.** | |  |
| --- | --- | --- | --- | --- | --- |
|  |  |  | **Lower** | **Upper** | **P Value** |
|  | FPlow | 3.845 | .394 | 37.545 | .247 |
|  | age | .959 | .862 | 1.067 | .440 |
|  | sex | .652 | .181 | 2.342 | .512 |
|  | CHRchronic | 1.060 | .693 | 1.620 | .789 |
|  | MDpolypharmacy | .469 | .127 | 1.740 | .258 |
|  | MOCASum | .930 | .792 | 1.092 | .374 |
|  | LOWEDU | 1.012 | .273 | 3.757 | .985 |
|  | CLASSICOH | 1.777 | .365 | 8.662 | .477 |
|  | Constant | 2.276 |  |  | .869 |

**(iii) Frail by phenotype treated high – Basic Logistic Regression Model**

|  | | **Odds Ratio** | **95% C.I. 95% C.I.** | |  |
| --- | --- | --- | --- | --- | --- |
|  |  |  | **Lower** | **Upper** | **P Value** |
|  | FPhigh | .000 | .000 | . | .998 |
|  | age | .982 | .892 | 1.082 | .719 |
|  | sex | .546 | .163 | 1.834 | .328 |
|  | Constant | .101 |  |  | .526 |

**(iv) Frail by phenotype treated high – Full Binary Logistic Regression Model**

|  | | **Odds Ratio** | **95% C.I. 95% C.I.** | |  |
| --- | --- | --- | --- | --- | --- |
|  |  |  | **Lower** | **Upper** | **P Value** |
|  | FPhigh | .000 | .000 | . | .998 |
|  | age | .972 | .879 | 1.076 | .587 |
|  | sex | .508 | .147 | 1.754 | .284 |
|  | CHRchronic | 1.108 | .734 | 1.671 | .626 |
|  | MDpolypharmacy | .650 | .195 | 2.174 | .485 |
|  | MOCASum | .930 | .795 | 1.087 | .361 |
|  | LOWEDU | .858 | .239 | 3.085 | .815 |
|  | CLASSICOH | 1.720 | .364 | 8.132 | .494 |
|  | Constant | 1.179 |  |  | .972 |

**(v) Non-frail by phenotype treated low – Basic Logistic Regression Model**

|  | | **Odds Ratio** | **95% C.I. 95% C.I.** | |  |
| --- | --- | --- | --- | --- | --- |
|  |  |  | **Lower** | **Upper** | **P Value** |
|  | NonFPlow | .962 | .253 | 3.658 | .954 |
|  | age | .974 | .881 | 1.077 | .608 |
|  | sex | .639 | .185 | 2.211 | .480 |
|  | Constant | .138 |  |  | .598 |

**(vi) Non-frail by phenotype treated low – Full Binary Logistic Regression Model**

|  | | **Odds Ratio** | **95% C.I. 95% C.I.** | |  |
| --- | --- | --- | --- | --- | --- |
|  |  |  | **Lower** | **Upper** | **P Value** |
|  | NonFPlow | 1.059 | .272 | 4.130 | .934 |
|  | age | .962 | .865 | 1.069 | .468 |
|  | sex | .598 | .168 | 2.130 | .428 |
|  | CHRchronic | 1.104 | .721 | 1.689 | .649 |
|  | MDpolypharmacy | .496 | .135 | 1.818 | .290 |
|  | MOCASum | .922 | .788 | 1.079 | .310 |
|  | LOWEDU | .993 | .266 | 3.707 | .992 |
|  | CLASSICOH | 1.915 | .390 | 9.391 | .423 |
|  | Constant | 2.337 |  |  | .863 |

**(vii) Non-frail by phenotype treated high – Basic Logistic Regression Model**

|  | | **Odds Ratio** | **95% C.I. 95% C.I.** | |  |
| --- | --- | --- | --- | --- | --- |
|  |  |  | **Lower** | **Upper** | **P Value** |
|  | NonFPhigh | .733 | .229 | 2.342 | .600 |
|  | age | .975 | .885 | 1.075 | .612 |
|  | sex | .545 | .162 | 1.830 | .326 |
|  | Constant | .206 |  |  | .669 |

**(viii) Non-frail by phenotype treated high – Full Binary Logistic Regression Model**

|  | | **Odds Ratio** | **95% C.I. 95% C.I.** | |  |
| --- | --- | --- | --- | --- | --- |
|  |  |  | **Lower** | **Upper** | **P Value** |
|  | NonFPhigh | .703 | .213 | 2.322 | .563 |
|  | age | .965 | .872 | 1.069 | .498 |
|  | sex | .510 | .148 | 1.756 | .286 |
|  | CHRchronic | 1.097 | .730 | 1.648 | .656 |
|  | MDpolypharmacy | .606 | .178 | 2.057 | .422 |
|  | MOCASum | .934 | .799 | 1.092 | .393 |
|  | LOWEDU | .846 | .237 | 3.016 | .796 |
|  | CLASSICOH | 1.786 | .372 | 8.586 | .469 |
|  | Constant | 2.294 |  |  | .862 |

**Outcome 4 : New heart failure diagnosis by Wave 2**

**(i) Frail by phenotype treated low – Basic Logistic Regression Model**

|  | | **Odds Ratio** | **95% C.I. 95% C.I.** | |  |
| --- | --- | --- | --- | --- | --- |
|  |  |  | **Lower** | **Upper** | **P Value** |
|  | FPlow | 5.422 | 1.122 | 26.204 | .035 |
|  | age | 1.083 | .999 | 1.173 | .054 |
|  | sex | .450 | .136 | 1.489 | .191 |
|  | Constant | .000 |  |  | .003 |

**(ii) Frailty by phenotype treated low – Full Binary Logistic Regresssion Model**

|  | | **Odds Ratio** | **95% C.I. 95% C.I.** | |  |
| --- | --- | --- | --- | --- | --- |
|  |  |  | **Lower** | **Upper** | **P Value** |
|  | FPlow | 4.604 | .893 | 23.736 | .068 |
|  | age | 1.081 | .986 | 1.185 | .095 |
|  | sex | .467 | .136 | 1.602 | .226 |
|  | CHRchronic | .873 | .594 | 1.283 | .490 |
|  | MDpolypharmacy | 5.172 | 1.092 | 24.502 | .038 |
|  | MOCASum | 1.009 | .872 | 1.167 | .902 |
|  | LOWEDU | .947 | .288 | 3.111 | .928 |
|  | CLASSICOH | .426 | .051 | 3.573 | .432 |
|  | Constant | .000 |  |  | .025 |

**(iii) Frail by phenotype treated high – Basic Logistic Regression Model**

|  | | **Odds Ratio** | **95% C.I. 95% C.I.** | |  |
| --- | --- | --- | --- | --- | --- |
|  |  |  | **Lower** | **Upper** | **P Value** |
|  | FPhigh | 1.455 | .173 | 12.246 | .730 |
|  | age | 1.086 | 1.002 | 1.177 | .045 |
|  | sex | .424 | .129 | 1.395 | .158 |
|  | Constant | .000 |  |  | .003 |

**(iv) Frail by phenotype treated high –Full Binary Logistic Regression Model**

|  | | **Odds Ratio** | **95% C.I 95% C.I.** | |  |
| --- | --- | --- | --- | --- | --- |
|  |  |  | **Lower** | **Upper** | **P Value** |
|  | FPhigh | 1.320 | .149 | 11.674 | .803 |
|  | age | 1.084 | .988 | 1.188 | .087 |
|  | sex | .435 | .126 | 1.494 | .186 |
|  | CHRchronic | .911 | .621 | 1.337 | .635 |
|  | MDpolypharmacy | 5.443 | 1.163 | 25.464 | .031 |
|  | MOCASum | .997 | .862 | 1.153 | .966 |
|  | LOWEDU | .922 | .279 | 3.047 | .893 |
|  | CLASSICOH | .494 | .060 | 4.057 | .512 |
|  | Constant | .000 |  |  | .027 |

**(v) Non-frail by phenotype treated low – Basic Logistic Regression Model**

|  | | **Odds Ratio** | **95% C.I. 95% C.I.** | |  |
| --- | --- | --- | --- | --- | --- |
|  |  |  | **Lower** | **Upper** | **P Value** |
|  | NonFPlow | .790 | .357 | 3.875 | 1.176 |
|  | age | .037 | 1.005 | 1.178 | 1.088 |
|  | sex | .162 | .130 | 1.405 | .428 |
|  | Constant | .002 |  |  | .000 |

**(vi) Non-frail by phenotype treated low – Full Binary Logistic Regression Model**

|  | | **Odds Ratio** | **95% C.I. 95% C.I..** | |  |
| --- | --- | --- | --- | --- | --- |
|  |  |  | **Lower** | **Upper** | **P Value** |
|  | NonFPlow | 1.035 | .311 | 3.445 | .955 |
|  | age | 1.083 | .988 | 1.187 | .087 |
|  | sex | .440 | .129 | 1.503 | .190 |
|  | CHRchronic | .905 | .618 | 1.325 | .608 |
|  | MDpolypharmacy | 5.531 | 1.178 | 25.982 | .030 |
|  | MOCASum | .996 | .863 | 1.150 | .960 |
|  | LOWEDU | .936 | .284 | 3.084 | .913 |
|  | CLASSICOH | .489 | .060 | 4.009 | .505 |
|  | Constant | .000 |  |  | .028 |

**(vii) Non-frail by phenotype treated high – Basic Logistic Regression Model**

**(viii) Non-frail by phenotype treated high – Full Binary Logistic Regression Model**

|  | | **Odds Ratio** | **95% C.I. 95% C.I.** | |  |
| --- | --- | --- | --- | --- | --- |
|  |  |  | **Lower** | **Upper** | **P Value** |
|  | NonFPhigh | .533 | .177 | 1.605 | .263 |
|  | age | 1.086 | 1.003 | 1.177 | .043 |
|  | sex | .426 | .130 | 1.401 | .160 |
|  | Constant | .000 |  |  | .005 |

|  | | **Odds Ratio** | **95% C.I. 95% C.I.** | |  |
| --- | --- | --- | --- | --- | --- |
|  |  |  | **Lower** | **Upper** | **P Value** |
|  | NonFPhigh | .619 | .201 | 1.906 | .403 |
|  | age | 1.086 | .991 | 1.191 | .078 |
|  | sex | .434 | .127 | 1.484 | .183 |
|  | CHRchronic | .900 | .614 | 1.320 | .590 |
|  | MDpolypharmacy | 5.234 | 1.115 | 24.576 | .036 |
|  | MOCASum | 1.002 | .866 | 1.158 | .981 |
|  | LOWEDU | .921 | .280 | 3.023 | .892 |
|  | CLASSICOH | .500 | .061 | 4.113 | .519 |
|  | Constant | .000 |  |  |  |

**Outcome 5 : Deceased by Wave 2**

**(i) Frail by phenotype treated low – Basic Logistic Regression Model**

|  | | **Odds Ratio** | **95% C.I. 95% C.I.** | |  |
| --- | --- | --- | --- | --- | --- |
|  |  |  | **Lower** | **Upper** | **P Value** |
|  | FPlow | 1.618 | .461 | 5.672 | .453 |
|  | age | 1.117 | 1.073 | 1.162 | .000 |
|  | sex | .963 | .548 | 1.692 | .895 |
|  | Constant | .000 |  |  | .000 |

**(ii) Frail by phenotype treated low – Full Binary Logistic Regression Model**

|  | | **Odds Ratio** | **95% C.I. 95% C.I.** | |  |
| --- | --- | --- | --- | --- | --- |
|  |  |  | **Lower** | **Upper** | **P Value** |
|  | FPlow | 1.025 | .283 | 3.715 | .970 |
|  | age | 1.079 | 1.029 | 1.131 | .002 |
|  | sex | .915 | .497 | 1.684 | .775 |
|  | CHRchronic | 1.181 | .973 | 1.434 | .092 |
|  | MDpolypharmacy | 1.764 | .905 | 3.437 | .095 |
|  | MOCASum | .878 | .818 | .942 | .000 |
|  | LOWEDU | .648 | .336 | 1.250 | .196 |
|  | CLASSICOH | .983 | .395 | 2.451 | .971 |
|  | Constant | .001 |  |  | .004 |

**(iii) Frail by phenotype treated high – Basic Logistic Regression Model**

|  | | **Odds Ratio** | **95% C.I. 95% C.I.** | |  |
| --- | --- | --- | --- | --- | --- |
|  |  |  | **Lower** | **Upper** | **P Value** |
|  | FPhigh | 2.060 | .806 | 5.261 | .131 |
|  | age | 1.105 | 1.060 | 1.152 | .000 |
|  | sex | .889 | .502 | 1.575 | .687 |
|  | Constant | .000 |  |  | .000 |

**(iv) Frail by phenotype treated high – Full binary Logistic Regression Model**

|  | | **Odds Ratio** | **95% C.I. 95% C.I.** | |  |
| --- | --- | --- | --- | --- | --- |
|  |  |  | **Lower** | **Upper** | **P Value** |
|  | FPhigh | 1.664 | .625 | 4.428 | .308 |
|  | age | 1.083 | 1.032 | 1.136 | .001 |
|  | sex | .791 | .431 | 1.452 | .449 |
|  | CHRchronic | 1.171 | .963 | 1.423 | .113 |
|  | MDpolypharmacy | 1.606 | .836 | 3.085 | .155 |
|  | MOCASum | .889 | .827 | .955 | .001 |
|  | LOWEDU | .549 | .282 | 1.067 | .077 |
|  | CLASSICOH | .805 | .304 | 2.132 | .662 |
|  | Constant | .001 |  |  | .003 |

**(v) Non-frail by phenotype treated low – Basic Regression Model**

|  | | **Odds Ratio** | **95% C.I. 95% C.I.** | |  |
| --- | --- | --- | --- | --- | --- |
|  |  |  | **Lower** | **Upper** | **P Value** |
|  | NonFPlow | .996 | .528 | 1.880 | .990 |
|  | age | 1.119 | 1.075 | 1.165 | .000 |
|  | sex | .956 | .546 | 1.676 | .876 |
|  | Constant | .000 |  |  | .000 |

**(vi) Non-frail by phenotype treated low – Full Binary Logistic Regression Model**

|  | | **Odds Ratio** | **95% C.I. 95% C.I.** | |  |
| --- | --- | --- | --- | --- | --- |
|  |  |  | **Lower** | **Upper** | **P Value** |
|  | NonFPlow | .880 | .450 | 1.723 | .710 |
|  | age | 1.078 | 1.028 | 1.131 | .002 |
|  | sex | .916 | .500 | 1.678 | .776 |
|  | CHRchronic | 1.181 | .975 | 1.430 | .088 |
|  | MDpolypharmacy | 1.780 | .913 | 3.469 | .090 |
|  | MOCASum | .878 | .818 | .941 | .000 |
|  | LOWEDU | .646 | .336 | 1.242 | .190 |
|  | CLASSICOH | .964 | .385 | 2.416 | .938 |
|  | Constant | .001 |  |  | .005 |

**(vii) Non-frail by phenotype treated high – Basic Regression Model**

|  | | **Odds Ratio** | **95% C.I. 95% C.I.** | |  |
| --- | --- | --- | --- | --- | --- |
|  |  |  | **Lower** | **Upper** | **P Value** |
|  | NonFPhigh | .498 | .281 | .885 | .017 |
|  | age | 1.111 | 1.066 | 1.157 | .000 |
|  | sex | .899 | .507 | 1.591 | .714 |
|  | Constant | .000 |  |  | .000 |

**(viii) Non-frail by phenotype treated high – Full Binary Logistic Regression Model**

|  | | **Odds Ratio** | **95% C.I. 95%C.I.** | |  |
| --- | --- | --- | --- | --- | --- |
|  |  |  | **Lower** | **Upper** | **P Value** |
|  | NonFPhigh | .626 | .341 | 1.149 | .130 |
|  | age | 1.086 | 1.036 | 1.138 | .001 |
|  | sex | .796 | .435 | 1.458 | .461 |
|  | CHRchronic | 1.163 | .958 | 1.413 | .127 |
|  | MDpolypharmacy | 1.540 | .800 | 2.967 | .196 |
|  | MOCASum | .893 | .830 | .960 | .002 |
|  | LOWEDU | .571 | .295 | 1.103 | .095 |
|  | CLASSICOH | .874 | .328 | 2.327 | .787 |
|  | Constant | .001 |  |  | .003 |

**Outcome 6 : Any falls/any fracture by Wave 2**

**(i) Frail by phenotype treated low – Basic Regression Model**

|  | | **Odds Ratio** | **95% C.I. 95% C.I.** | |  |
| --- | --- | --- | --- | --- | --- |
|  |  |  | **Lower** | **Upper** | **P Value** |
|  | FPlow | 2.429 | 1.176 | 5.015 | .016 |
|  | age | 1.016 | .995 | 1.037 | .137 |
|  | sex | 1.698 | 1.308 | 2.205 | .000 |
|  | Constant | .057 |  |  | .000 |

**(ii) Frail by phenotype treated low – Full Binary Logistic Regression Model**

|  | | **Odds Ratio** | **95% C.I. 95% C.I.** | |  |
| --- | --- | --- | --- | --- | --- |
|  |  |  | **Lower** | **Upper** | **P Value** |
|  | FPhigh | .890 | 3.978 | .098 | 1.881 |
|  | age | .991 | .098 | .228 | 1.014 |
|  | sex | 1.134 | .228 | .004 | 1.487 |
|  | CHRchronic | 1.106 | .004 | .000 | 1.215 |
|  | MDpolypharmacy | .816 | .000 | .595 | 1.079 |
|  | MOCASum | .956 | .595 | .644 | .991 |
|  | LOWEDU | .631 | .644 | .248 | .843 |
|  | CLASSICOH | 1.145 | .248 | .008 | 1.687 |
|  | Constant |  | .008 | .007 | .054 |

**(iii) Frail by phenotype treated high – Basic Regression Model**

|  | | **Odds Ratio** | **95% C.I. 95% C.I.** | |  |
| --- | --- | --- | --- | --- | --- |
|  |  |  | **Lower** | **Upper** | **P Value** |
|  | FPhigh | 1.445 | .731 | 2.859 | .290 |
|  | age | 1.018 | .997 | 1.040 | .086 |
|  | sex | 1.708 | 1.317 | 2.215 | .000 |
|  | Constant | .048 |  |  | .000 |

**(iv)Frail by phenotype treated high – Full Binary Logistic Regression Model**

|  | | **Odds Ratio** | **95% C.I. 95% C.I.** | |  |
| --- | --- | --- | --- | --- | --- |
|  |  |  | **Lower** | **Upper** | **P Value** |
|  | FPhigh | 1.213 | .600 | 2.452 | .591 |
|  | age | 1.014 | .992 | 1.038 | .213 |
|  | sex | 1.496 | 1.143 | 1.959 | .003 |
|  | CHRchronic | 1.214 | 1.105 | 1.332 | .000 |
|  | MDpolypharmacy | 1.077 | .817 | 1.420 | .598 |
|  | MOCASum | .980 | .945 | 1.017 | .284 |
|  | LOWEDU | .838 | .627 | 1.120 | .232 |
|  | CLASSICOH | 1.689 | 1.146 | 2.490 | .008 |
|  | Constant | .070 |  |  | .014 |

**(v) Non-frail by phenotype treated low – Basic Regression Model**

|  | | **Odds Ratio** | **95% C.I. 95% C.I.** | |  |
| --- | --- | --- | --- | --- | --- |
|  |  |  | **Lower** | **Upper** | **P Value** |
|  | NonFPlow | .733 | .546 | .984 | .039 |
|  | age | 1.016 | .995 | .039 | .139 |
|  | sex | 1.660 | 1.278 | .139 | .000 |
|  | Constant | .067 |  | .000 | .001 |

**(vi) Non-frail by phenotype treated low – Full Binary Logistic Regression Model**

|  | | **Odds Ratio** | **95% C.I. 95% C.I.** | |  |
| --- | --- | --- | --- | --- | --- |
|  |  |  | **Lower** | **Upper** | **P Value** |
|  | NonFPlow | .717 | .530 | .971 | .031 |
|  | age | 1.012 | .990 | 1.035 | .286 |
|  | sex | 1.479 | 1.128 | 1.939 | .005 |
|  | CHRchronic | 1.224 | 1.114 | 1.344 | .000 |
|  | MDpolypharmacy | 1.130 | .854 | 1.494 | .392 |
|  | MOCASum | .987 | .952 | 1.023 | .477 |
|  | LOWEDU | .831 | .622 | 1.112 | .213 |
|  | CLASSICOH | 1.626 | 1.100 | 2.404 | .015 |
|  | Constant | .074 |  |  | .017 |

**(vii) Non-frail treated high – Basic Regression Model**

|  | | **Odds Ratio** | **95% C.I. 95% C.I.** | |  |
| --- | --- | --- | --- | --- | --- |
|  |  |  | **Lower** | **Upper** | **P Value** |
|  | NonFPhigh | .999 | .764 | 1.305 | .994 |
|  | age | 1.020 | 1.000 | 1.041 | .055 |
|  | sex | 1.673 | 1.290 | 2.171 | .000 |
|  | Constant | .044 |  |  | .000 |

**(viii) Non-frail treated high – Full binary Logistic Regression Model**

|  | | **Odds Ratio** | **95% C.I. 95% C.I.** | |  |
| --- | --- | --- | --- | --- | --- |
|  |  |  | **Lower** | **Upper** | **P Value** |
|  | NonFPhigh | 1.075 | .814 | 1.420 | .612 |
|  | age | 1.015 | .993 | 1.038 | .178 |
|  | sex | 1.492 | 1.140 | 1.954 | .004 |
|  | CHRchronic | 1.217 | 1.109 | 1.336 | .000 |
|  | MDpolypharmacy | 1.091 | .826 | 1.441 | .541 |
|  | MOCASum | .979 | .944 | 1.016 | .261 |
|  | LOWEDU | .843 | .631 | 1.126 | .248 |
|  | CLASSICOH | 1.684 | 1.141 | 2.485 | .009 |
|  | Constant | .063 |  |  | .011 |

**Outcome 7: TIA or Stroke by Wave 2**

**(i) Frail by phenotype treated low – Basic Regression Model**

|  | | **Odds Ratio** | **95% C.I. 95% C.I.** | |  |
| --- | --- | --- | --- | --- | --- |
|  |  |  | **Lower** | **Upper** | **P Value** |
|  | FPlow | .897 | .118 | 6.828 | .917 |
|  | age | 1.025 | .974 | 1.079 | .337 |
|  | sex | .505 | .249 | 1.023 | .058 |
|  | Constant | .014 |  |  | .028 |

**(ii) Frail by phenotype treated low – Full Binary Logistic Regression Model**

|  | | **Odds Ratio** | **95% C.I. 95% C.I.** | |  |
| --- | --- | --- | --- | --- | --- |
|  |  |  | **Lower** | **Upper** | **P Value** |
|  | FPlow | .500 | .062 | 4.031 | .515 |
|  | age | 1.009 | .952 | 1.069 | .759 |
|  | sex | .449 | .213 | .946 | .035 |
|  | CHRchronic | .972 | .776 | 1.217 | .803 |
|  | MDpolypharmacy | 2.690 | 1.221 | 5.925 | .014 |
|  | MOCASum | .942 | .860 | 1.032 | .197 |
|  | LOWEDU | .732 | .340 | 1.574 | .424 |
|  | CLASSICOH | 3.797 | 1.755 | 8.216 | .001 |
|  | Constant | .102 |  |  | .419 |

**(iii) Frail by phenotype treated high – Basic Regression Model**

|  | | **Odds Ratio** | **95% C.I. 95% C.I.** | |  |
| --- | --- | --- | --- | --- | --- |
|  |  |  | **Lower** | **Upper** | **P Value** |
|  | FPlow | 2.112 | .593 | 7.527 | .249 |
|  | age | 1.033 | .982 | 1.086 | .206 |
|  | sex | .521 | .263 | 1.032 | .062 |
|  | Constant | .008 |  |  | .012 |

**(iv) Frail by phenotype treated high – Full Binary Logistic Regression Model**

|  | | **Odds Ratio** | **95% C.I. 95% C.I.** | |  |
| --- | --- | --- | --- | --- | --- |
|  |  |  | **Lower** | **Upper** | **P Value** |
|  | FPhigh | 1.622 | .443 | 5.940 | .465 |
|  | age | 1.017 | .961 | 1.076 | .555 |
|  | sex | .473 | .231 | .971 | .041 |
|  | CHRchronic | .977 | .787 | 1.212 | .832 |
|  | MDpolypharmacy | 2.376 | 1.114 | 5.070 | .025 |
|  | MOCASum | .937 | .859 | 1.022 | .141 |
|  | LOWEDU | .799 | .382 | 1.670 | .551 |
|  | CLASSICOH | 3.444 | 1.617 | 7.335 | .001 |
|  | Constant | .064 |  |  | .311 |

**(v) Non-frail by phenotype treated low – Basic Regression Model**

|  | | **Odds Ratio** | **95% C.I. 95% C.I.** | |  |
| --- | --- | --- | --- | --- | --- |
|  |  |  | **Lower** | **Upper** | **P Value** |
|  | NonFPlow | .392 | .151 | 1.019 | .055 |
|  | age | 1.023 | .972 | 1.076 | .382 |
|  | sex | .516 | .254 | 1.047 | .067 |
|  | Constant | .019 |  |  | .042 |

**(vi) Non-frail by phenotype treated low – Full Binary Logistic Regression Models**

|  | | **Odds Ratio** | **95% C.I. 95% C.I.** | |  |
| --- | --- | --- | --- | --- | --- |
|  |  |  | **Lower** | **Upper** | **P Value** |
|  | NonFPlow | .403 | .153 | 1.065 | .067 |
|  | age | 1.007 | .951 | 1.066 | .816 |
|  | sex | .461 | .220 | .969 | .041 |
|  | CHRchronic | .956 | .765 | 1.194 | .689 |
|  | MDpolypharmacy | 2.864 | 1.295 | 6.335 | .009 |
|  | MOCASum | .945 | .864 | 1.033 | .215 |
|  | LOWEDU | .757 | .354 | 1.620 | .473 |
|  | CLASSICOH | 3.363 | 1.557 | 7.265 | .002 |
|  | Constant | .131 |  |  | .464 |

**(vii) Non-frail treated high – Basic Logistic Regression Model**

|  | | **Odd Ratio** | **95% C.I. 95% C.I.** | |  |
| --- | --- | --- | --- | --- | --- |
|  |  |  | **Lower** | **Upper** | **P Value** |
|  | NonFPhigh | 1.512 | .740 | 3.091 | .257 |
|  | age | 1.041 | .991 | 1.093 | .108 |
|  | sex | .521 | .263 | 1.032 | .062 |
|  | Constant | .003 |  |  | .003 |

**(viii) Non-frail treated high – Full Binary Logistic Regression Model**

|  | | **Odds Ratio** | **95% C.I. 95% C.I.** | |  |
| --- | --- | --- | --- | --- | --- |
|  |  |  | **Lower** | **Upper** | **P Value** |
|  | NonFPhigh | 1.678 | .800 | 3.520 | .171 |
|  | age | 1.021 | .967 | 1.079 | .453 |
|  | sex | .457 | .222 | .941 | .034 |
|  | CHRchronic | .987 | .795 | 1.225 | .903 |
|  | MDpolypharmacy | 2.627 | 1.226 | 5.630 | .013 |
|  | MOCASum | .931 | .854 | 1.016 | .111 |
|  | LOWEDU | .807 | .384 | 1.694 | .571 |
|  | CLASSICOH | 3.408 | 1.605 | 7.236 | .001 |
|  | Constant | .038 |  |  | .222 |
