# Supplementary material for: Do the frail experience more adverse events from intensive blood pressure control? A 2-year prospective study in the Irish Longitudinal Study on Ageing (TILDA)
Source: eClinicalMedicine. 2022 Feb 19;45:101304. doi: 10.1016/j.eclinm.2022.101304 (PMC8860911; doi:10.1016/j.eclinm.2022.101304)
Supplement: Supplementary file 2 [file mmc2.docx]

**Appendix 2. Binary Logistic Regression Analysis for outcomes at Wave 2 for 4 Groups with Frailty by Clinical Frailty Score (CFS)**

**Index :**

Outcome 1 Hospitalisation: Page 2-5

Outcome 2 Syncope: Page 6-9

Outcome 3 Heart Attack: Page 10-13

Outcome 4 Heart Failure: Page 14-17

Outcome 5 Deceased by Waave 2: Page 18-21

Ouctome 6 Falls or Fractures: Page 22-25

Outcome 7 TIA or Stroke: Page 26-29

**Abbreviations/Legend:**

FRAILCFStreatedlow **–** Frail by CFS ( Clinical Frailty Scale with blood pressure treated low)

FRAILCFStreatedhigh – Frail by CFS with blood pressure treated high

NONFRAILCFStreatedlow – Non-frail by CFS with blood pressure treated low

NONFRAILCFStreatedhigh – Non-frail by CFS with blood pressure treated high

CHRchronic **-** Number of chronic medical conditions, counted from the following list: heart attack or heart failure or angina, cataracts, hypertension, high cholesterol, stroke, diabetes, lung disease, asthma, arthritis,

MDpolypharmacy **-** Polypharmacy, defined as a participant being on 5 or more regular medications.

MOCASum - Montreal Cognitive Assessment (MOCA) score

LOWEDU - Low level of education, defined as educated up to primary school level.

CLASSICOH **-** defined as a drop of ≥20 mmHg in SBP and/or ≥10 mmHg DBP on standing from a seated position, which was also measured in TILDA as described elsewhere

**Outcome 1. Hospitalisation by Wave 2**

**(i) Frail CFS treated low – Basic Logistic Regression Model**

|  | | **Odds Ratio** | **95% C.I. 95% C.I.** | |  |
| --- | --- | --- | --- | --- | --- |
|  |  |  | **Lower** | **Upper** | **P Value** |
|  | FRAILCFStreatedlow | 1.922 | 1.209 |  | .006 |
|  | age | 1.030 | 1.010 | .006 | .003 |
|  | sex | .629 | .492 | .003 | .000 |
|  | Constant | .067 |  | .000 | .000 |

**(ii) Frail CFS treated low – Full Binary Logistic Regression Model**

|  | | **Odds Ratio** | **95% C.I. 95% C.I.** | |  |
| --- | --- | --- | --- | --- | --- |
|  |  |  | **Lower** | **Upper** | **P Value** |
|  | FRAILCFStreatedlow | 1.422 | .872 | 2.319 | .158 |
|  | age | 1.025 | 1.001 | 1.049 | .040 |
|  | sex | .576 | .431 | .771 | .000 |
|  | CHRchronic | 1.125 | 1.021 | 1.240 | .018 |
|  | MDpolypharmacy | 1.649 | 1.224 | 2.220 | .001 |
|  | MOCASum | .961 | .925 | .998 | .039 |
|  | LOWEDU | .978 | .722 | 1.324 | .885 |
|  | CLASSICOH | 1.232 | .809 | 1.878 | .331 |
|  | Constant | .153 |  |  | .095 |

**(iii) Frail by CFS treated high - Basic Logistic Regression Model**

|  | | **Odds Ratio** | **95% C.I. 95% C.I.** | |  |
| --- | --- | --- | --- | --- | --- |
|  |  |  | **Lower** | **Upper** | **P Value** |
|  | FRAILCFStreatedhigh | 1.532 | 1.061 | 2.212 | .023 |
|  | age | 1.028 | 1.009 | 1.048 | .005 |
|  | sex | .638 | .500 | .815 | .000 |
|  | Constant | .072 |  |  | .000 |

(**iv) Frail by CFS treated high – Full Binary Logistic Regression Model**

|  | | **Odds Ratio** | **95% C.I. 95% C.I.** | |  |
| --- | --- | --- | --- | --- | --- |
|  |  |  | **Lower** | **Upper** | **P Value** |
|  | FRAILCFStreatedhigh | 1.276 | .862 | 1.887 | .223 |
|  | age | 1.024 | 1.000 | 1.048 | .047 |
|  | sex | .580 | .433 | .775 | .000 |
|  | CHRchronic | 1.129 | 1.024 | 1.244 | .015 |
|  | MDpolypharmacy | 1.675 | 1.246 | 2.252 | .001 |
|  | MOCASum | .962 | .926 | .999 | .043 |
|  | LOWEDU | .964 | .712 | 1.306 | .815 |
|  | CLASSICOH | 1.194 | .782 | 1.824 | .411 |
|  | Constant | .155 |  |  | .098 |

**(v) Non-frail by CFS treated low - Basic Logistic Regression Model**

|  | | **Odds Ratio** | **95% C.I. 95% C.I.** | |  |
| --- | --- | --- | --- | --- | --- |
|  |  |  | **Lower** | **Upper** | **P Value** |
|  | NONFRAILCFStreatedlow | .964 | .703 | 1.320 | .817 |
|  | age | 1.035 | 1.014 | 1.056 | .001 |
|  | sex | .650 | .497 | .849 | .002 |
|  | Constant | .048 |  |  | .000 |

**(vi) Non-frail by CFS treated low – Non-frail by CFS treated high**

|  | | **Odds Ratio** | **95% C.I. 95% C.I.** | |  |
| --- | --- | --- | --- | --- | --- |
|  |  |  | **Lower** | **Upper** | **P Value** |
|  | NONFRAILCFStreatedlow | .928 | .671 | 1.282 | .649 |
|  | age | 1.025 | 1.001 | 1.049 | .037 |
|  | sex | .579 | .433 | .774 | .000 |
|  | CHRchronic | 1.133 | 1.029 | 1.248 | .011 |
|  | MDpolypharmacy | 1.694 | 1.260 | 2.278 | .000 |
|  | MOCASum | .959 | .923 | .995 | .028 |
|  | LOWEDU | .968 | .715 | 1.310 | .832 |
|  | CLASSICOH | 1.213 | .795 | 1.850 | .370 |
|  | Constant | .160 |  |  | .105 |

**(vii) Non-frail by CFS treated high - Basic Logistic Regression Model**

|  | | **Odds Ratio** | **95% C.I. 95% C.I.** | |  |
| --- | --- | --- | --- | --- | --- |
|  |  |  | **Lower** | **Upper** | **P Value** |
|  | NONFRAILCFStreatedhigh | .748 | .571 | .979 | .034 |
|  | age | 1.032 | 1.011 | 1.053 | .003 |
|  | sex | .641 | .490 | .839 | .001 |
|  | Constant | .069 |  |  | .001 |

**(viii) Non-frail by CFS treated high - Non-frail by CFS treated high**

|  | | **Odds Ratio** | **95% C.I. 95% C.I.** | |  |
| --- | --- | --- | --- | --- | --- |
|  |  |  | **Lower** | **Upper** | **P Value** |
|  | NONFRAILCFStreatedhigh | .830 | .622 | 1.107 | .205 |
|  | age | 1.025 | 1.002 | 1.049 | .037 |
|  | sex | .578 | .433 | .773 | .000 |
|  | CHRchronic | 1.128 | 1.024 | 1.243 | .015 |
|  | MDpolypharmacy | 1.645 | 1.221 | 2.217 | .001 |
|  | MOCASum | .962 | .926 | 1.000 | .048 |
|  | LOWEDU | .968 | .715 | 1.311 | .835 |
|  | CLASSICOH | 1.232 | .808 | 1.878 | .333 |
|  | Constant | .162 |  |  | .106 |

**Outcome 2 : Syncope by Wave 2**

**(i) Frail CFS treated low – Basic Logistic Regression Model**

|  | | **Odds Ratio** | **95% C.I. 95% C.I.** | |  |
| --- | --- | --- | --- | --- | --- |
|  |  |  | **Lower** | **Upper** | **P Value** |
|  | FRAILCFStreatedlow | 2.245 | .918 | 5.490 | .076 |
|  | age | 1.014 | .971 | 1.060 | .521 |
|  | sex | 1.079 | .616 | 1.889 | .790 |
|  | Constant | .013 |  |  | .010 |

**(ii) Frail CFS treated low – Full Binary Logistic Regression Model**

|  | | **Odds Ratio** | **95% C.I. 95% C.I.** | |  |
| --- | --- | --- | --- | --- | --- |
|  |  |  | **Lower** | **Upper** | **P Value** |
|  | FRAILCFStreatedlow | 2.143 | .830 | 5.533 | .115 |
|  | age | 1.035 | .984 | 1.089 | .186 |
|  | sex | 1.449 | .764 | 2.747 | .257 |
|  | CHRchronic | .903 | .720 | 1.132 | .377 |
|  | MDpolypharmacy | 1.467 | .753 | 2.859 | .261 |
|  | MOCASum | 1.073 | .986 | 1.168 | .103 |
|  | LOWEDU | 1.690 | .880 | 3.247 | .115 |
|  | CLASSICOH | 1.485 | .648 | 3.401 | .350 |
|  | Constant | .000 |  |  | .001 |

**(iii) Frail by CFS treated high - Basic Logistic Regression Model**

|  | | **Odds Ratio** | **95% C.I. 95% C.I.** | |  |
| --- | --- | --- | --- | --- | --- |
|  |  |  | **Lower** | **Upper** | **P Value** |
|  | FRAILCFStreatedhigh | 1.526 | .662 | 3.519 | .321 |
|  | age | 1.013 | .970 | 1.059 | .556 |
|  | sex | 1.118 | .640 | 1.954 | .695 |
|  | Constant | .013 |  |  | .011 |

**(iv) Frail by CFS treated high – Full Binary Logistic Regression Model**

|  | | **Odds Ratio** | **95% C.I. 95% C.I.** | |  |
| --- | --- | --- | --- | --- | --- |
|  |  |  | **Lower** | **Upper** | **P Value** |
|  | FRAILCFStreatedhigh | 1.099 | .436 | 2.767 | .842 |
|  | age | 1.035 | .984 | 1.089 | .186 |
|  | sex | 1.484 | .784 | 2.809 | .226 |
|  | CHRchronic | .918 | .733 | 1.149 | .456 |
|  | MDpolypharmacy | 1.562 | .808 | 3.020 | .185 |
|  | MOCASum | 1.067 | .980 | 1.162 | .136 |
|  | LOWEDU | 1.627 | .848 | 3.121 | .143 |
|  | CLASSICOH | 1.444 | .630 | 3.312 | .385 |
|  | Constant | .000 |  |  | .001 |

**(v) Non-frail by CFS treated low - Basic Logistic Regression Model**

|  | | **Odds Ratio** | **95% C.I. 95% C.I.** | |  |
| --- | --- | --- | --- | --- | --- |
|  |  |  | **Lower** | **Upper** | **P Value** |
|  | NONFRAILCFStreatedlow | .452 | .189 | 1.080 | .074 |
|  | age | 1.011 | .968 | 1.056 | .625 |
|  | sex | 1.247 | .693 | 2.243 | .461 |
|  | Constant | .019 |  |  | .019 |

**(vi) Non-frail by CFS treated low - Full Binary Logistic Regression Model**

|  | | **Odds Ratio** | **95% C.I. 95% C.I.** | |  |
| --- | --- | --- | --- | --- | --- |
|  |  |  | **Lower** | **Upper** | **P Value** |
|  | NONFRAILCFStreatedlow | .455 | .188 | 1.104 | .082 |
|  | age | 1.032 | .981 | 1.085 | .222 |
|  | sex | 1.499 | .788 | 2.850 | .217 |
|  | CHRchronic | .905 | .723 | 1.133 | .384 |
|  | MDpolypharmacy | 1.633 | .842 | 3.167 | .147 |
|  | MOCASum | 1.067 | .980 | 1.161 | .135 |
|  | LOWEDU | 1.655 | .860 | 3.186 | .131 |
|  | CLASSICOH | 1.349 | .589 | 3.092 | .479 |
|  | Constant | .001 |  |  | .002 |

**(vii) Non-frail by CFS treated high - Basic Logistic Regression Model**

|  | | **Odds Ratio** | **95% C.I. 95% C.I.** | |  |
| --- | --- | --- | --- | --- | --- |
|  |  |  | **Lower** | **Upper** | **P Value** |
|  | NONFRAILCFStreatedhigh | 1.094 | .607 | 1.970 | .765 |
|  | age | 1.016 | .972 | 1.062 | .492 |
|  | sex | 1.253 | .697 | 2.250 | .451 |
|  | Constant | .011 |  |  | .010 |

**(viii) Non-frail by CFS treated high- Full Binary Logistic Regression Model**

|  | | **Odds Ratio** | **95% C.I. 95% C.I.** | |  |
| --- | --- | --- | --- | --- | --- |
|  |  |  | **Lower** | **Upper** | **P Value** |
|  | NONFRAILCFStreatedhigh | 1.208 | .637 | 2.292 | .562 |
|  | age | 1.036 | .985 | 1.089 | .171 |
|  | sex | 1.485 | .784 | 2.813 | .225 |
|  | CHRchronic | .922 | .737 | 1.154 | .480 |
|  | MDpolypharmacy | 1.611 | .828 | 3.134 | .160 |
|  | MOCASum | 1.062 | .976 | 1.157 | .163 |
|  | LOWEDU | 1.625 | .846 | 3.120 | .145 |
|  | CLASSICOH | 1.445 | .632 | 3.302 | .383 |
|  | Constant | .000 |  |  | .001 |

**Outcome 3 : Heart Attack by Wave 2**

**(i) Frail CFS treated low – Basic Logistic Regression Model**

|  | | **Odds Ratio** | **95% C.I. 95% C.I.** | |  |
| --- | --- | --- | --- | --- | --- |
|  |  |  | **Lower** | **Upper** | **P Value** |
|  | FRAILCFStreatedlow | 2.994 | .656 | 13.658 | .157 |
|  | age | .933 | .850 | 1.024 | .142 |
|  | sex | .641 | .230 | 1.784 | .394 |
|  | Constant | 3.117 |  |  | .738 |

**(ii) Frail CFS treated low – Full Binary Logistic Regression Model**

|  | | **Odds Ratio** | **95% C.I 95% C.I.** | |  |
| --- | --- | --- | --- | --- | --- |
|  |  |  | **Lower** | **Upper** | **P Value** |
|  | FRAILCFStreatedlow | 3.559 | .661 | 19.176 | .140 |
|  | age | .960 | .866 | 1.065 | .444 |
|  | sex | .501 | .145 | 1.731 | .275 |
|  | CHRchronic | 1.058 | .697 | 1.604 | .792 |
|  | MDpolypharmacy | .546 | .155 | 1.921 | .346 |
|  | MOCASum | .938 | .803 | 1.096 | .421 |
|  | LOWEDU | .859 | .241 | 3.070 | .815 |
|  | CLASSICOH | 1.839 | .385 | 8.782 | .445 |
|  | Constant | 2.436 |  |  | .854 |

**(iii) Frail by CFS treated high - Basic Logistic Regression Model**

|  | | **Odds Ratio** | **95% C.I. 95% C.I.** | |  |
| --- | --- | --- | --- | --- | --- |
|  |  |  | **Lower** | **Upper** | **P Value** |
|  | FRAILCFStreatedhigh | .665 | .086 | 5.131 | .695 |
|  | age | .940 | .857 | 1.031 | .189 |
|  | sex | .653 | .235 | 1.814 | .414 |
|  | Constant | 1.977 |  |  | .840 |

**(iv) Frail by CFS treated high – Full Binary Logistic Regression Model**

|  | | **Odds Ratio** | **95% C.I. 95% C.I.** | |  |
| --- | --- | --- | --- | --- | --- |
|  |  |  | **Lower** | **Upper** | **P Value** |
|  | FRAILCFStreatedhigh | .534 | .064 | 4.464 | .562 |
|  | age | .969 | .875 | 1.073 | .543 |
|  | sex | .504 | .146 | 1.741 | .279 |
|  | CHRchronic | 1.118 | .740 | 1.689 | .596 |
|  | MDpolypharmacy | .646 | .192 | 2.174 | .481 |
|  | MOCASum | .926 | .792 | 1.084 | .338 |
|  | LOWEDU | .861 | .242 | 3.067 | .817 |
|  | CLASSICOH | 1.765 | .371 | 8.401 | .475 |
|  | Constant | 1.688 |  |  | .912 |

**(v) Non-frail by CFS treated low - Basic Logistic Regression Model**

|  | | **Odds Ratio** | **95% C.I. 95% C.I.** | |  |
| --- | --- | --- | --- | --- | --- |
|  |  |  | **Lower** | **Upper** | **P Value** |
|  | NONFRAILCFStreatedlow | .881 | .243 | 3.187 | .847 |
|  | age | .963 | .880 | 1.053 | .405 |
|  | sex | .795 | .272 | 2.321 | .674 |
|  | Constant | .289 |  |  | .710 |

**(vi)** **Non-frail by CFS treated low - Full Binary Logistic Regression Model**

|  | | **Odds Ratio** | **95% C.I. 95% C.I.** | |  |
| --- | --- | --- | --- | --- | --- |
|  |  |  | **Lower** | **Upper** | **P Value** |
|  | NONFRAILCFStreatedlow | 1.036 | .276 | 3.895 | .958 |
|  | age | .966 | .873 | 1.069 | .499 |
|  | sex | .510 | .148 | 1.757 | .286 |
|  | CHRchronic | 1.105 | .734 | 1.661 | .633 |
|  | MDpolypharmacy | .634 | .188 | 2.133 | .461 |
|  | MOCASum | .933 | .800 | 1.089 | .378 |
|  | LOWEDU | .853 | .240 | 3.038 | .807 |
|  | CLASSICOH | 1.704 | .358 | 8.121 | .503 |
|  | Constant | 1.748 |  |  | .906 |

**(vii) Non-frail by CFS treated high - Basic Logistic Regression Model**

|  | | **Odds Ratio** | **95% C.I. 95% C.I.** | |  |
| --- | --- | --- | --- | --- | --- |
|  |  |  | **Lower** | **Upper** | **P Value** |
|  | NONFRAILCFStreatedhigh | .656 | .223 | 1.933 | .444 |
|  | age | .958 | .874 | 1.049 | .354 |
|  | sex | .772 | .264 | 2.259 | .637 |
|  | Constant | .512 |  |  | .846 |

**(viii) Non-frail by CFS treated high- Full Binary Logistic Regression Model**

|  | | **Odds Ratio** | **95% C.I. 95% C.I.** | |  |
| --- | --- | --- | --- | --- | --- |
|  |  |  | **Lower** | **Upper** | **P Value** |
|  | NONFRAILCFStreatedhigh | .777 | .233 | 2.591 | .682 |
|  | age | .964 | .870 | 1.067 | .478 |
|  | sex | .510 | .148 | 1.755 | .286 |
|  | CHRchronic | 1.093 | .726 | 1.646 | .669 |
|  | MDpolypharmacy | .611 | .179 | 2.083 | .431 |
|  | MOCASum | .937 | .802 | 1.095 | .416 |
|  | LOWEDU | .854 | .241 | 3.032 | .807 |
|  | CLASSICOH | 1.734 | .365 | 8.241 | .489 |
|  | Constant | 2.168 |  |  | .871 |

**Outcome 4 : New diagnosis of Heart Failure by Wave 2**

**(i) Frail by CFS treated low – Basic Logistic Regression Model**

|  | | **Odds Ratio** | **95% C.I. 95% C.I.** | |  |
| --- | --- | --- | --- | --- | --- |
|  |  |  | **Lower** | **Upper** | **P Value** |
|  | FRAILCFStreatedlow | .000 | .000 | . | .997 |
|  | age | 1.068 | .993 | 1.149 | .075 |
|  | sex | .431 | .149 | 1.252 | .122 |
|  | Constant | .000 |  |  | .004 |

**(ii) Frail by CFS treated low –Full Binary Logistic Regression Model**

|  | | **Odds Ratio** | **95% C.I. 95% C.I.** | |  |
| --- | --- | --- | --- | --- | --- |
|  |  |  | **Lower** | **Upper** | **P Value** |
|  | FRAILCFStreatedlow | .000 | .000 | . | .997 |
|  | age | 1.086 | .991 | 1.189 | .076 |
|  | sex | .429 | .125 | 1.467 | .177 |
|  | CHRchronic | .939 | .640 | 1.379 | .749 |
|  | MDpolypharmacy | 5.939 | 1.268 | 27.819 | .024 |
|  | MOCASum | .987 | .855 | 1.141 | .862 |
|  | LOWEDU | .868 | .261 | 2.888 | .818 |
|  | CLASSICOH | .484 | .060 | 3.934 | .497 |
|  | Constant | .000 |  |  | .027 |

**(iii) Frail by CFS treated high -Basic Logistic Regression Model**

|  | | **Odds Ratio** | **95% C.I. 95%C.I.** | |  |
| --- | --- | --- | --- | --- | --- |
|  |  |  | **Lower** | **Upper** | **P Value** |
|  | FRAILCFStreatedhigh | 2.496 | .773 | 8.058 | .126 |
|  | age | 1.056 | .980 | 1.137 | .151 |
|  | sex | .421 | .145 | 1.223 | .112 |
|  | Constant | .001 |  |  | .009 |

**(iv) Frail by CFS treated high – Full Binary Logistic Regression Model**

|  | | **Odds Ratio** | **95% C.I. 95% C.I.** | |  |
| --- | --- | --- | --- | --- | --- |
|  |  |  | **Lower** | **Upper** | **P Value** |
|  | FRAILCFStreatedhigh | 2.325 | .660 | 8.191 | .189 |
|  | age | 1.076 | .981 | 1.179 | .121 |
|  | sex | .427 | .125 | 1.455 | .174 |
|  | CHRchronic | .887 | .607 | 1.298 | .538 |
|  | MDpolypharmacy | 5.343 | 1.137 | 25.101 | .034 |
|  | MOCASum | 1.011 | .874 | 1.171 | .880 |
|  | LOWEDU | .985 | .297 | 3.269 | .980 |
|  | CLASSICOH | .443 | .054 | 3.669 | .451 |
|  | Constant | .000 |  |  | .030 |

**(v) Non-frail by CFS treated low –Basic Logistic Regression Model**

|  | | **Odds Ratio** | **95% C.I. 95% C.I.** | |  |
| --- | --- | --- | --- | --- | --- |
|  |  |  | **Lower** | **Upper** | **P Value** |
|  | NONFRAILCFStreatedlow | 2.007 | .720 | 5.591 | .183 |
|  | age | 1.104 | 1.030 | 1.184 | .005 |
|  | sex | .365 | .126 | 1.053 | .062 |
|  | Constant | .000 |  |  | .000 |

**(vi) Non -frail by CFS treated low – Full Binary Logistic Regression Model**

|  | | **Odds Ratio** | **95% C.I. 95% C.I.** | |  |
| --- | --- | --- | --- | --- | --- |
|  |  |  | **Lower** | **Upper** | **P Value** |
|  | NONFRAILCFStreatedlow | 2.740 | .885 | 8.480 | .080 |
|  | age | 1.096 | .998 | 1.204 | .056 |
|  | sex | .420 | .123 | 1.440 | .168 |
|  | CHRchronic | .921 | .627 | 1.353 | .675 |
|  | MDpolypharmacy | 5.479 | 1.167 | 25.722 | .031 |
|  | MOCASum | .995 | .860 | 1.151 | .947 |
|  | LOWEDU | .863 | .261 | 2.852 | .810 |
|  | CLASSICOH | .511 | .062 | 4.239 | .534 |
|  | Constant | .000 |  |  | .016 |

**(vii) Non-frail by CFS treated high – Basic Logistic Regression Model**

|  | | **Odds Ratio** | **95% C.I. 95% C.I.** | |  |
| --- | --- | --- | --- | --- | --- |
|  |  |  | **Lower** | **Upper** | **P Value** |
|  | NONFRAILCFStreatedhigh | .238 | .067 | .841 | .026 |
|  | age | 1.085 | 1.013 | 1.164 | .021 |
|  | sex | .331 | .114 | .960 | .042 |
|  | Constant | .000 |  |  | .002 |

**(viii) Non-frail by CFS treated high – Full Binary Logistic Regression Model**

|  | **Odds Ratio** | **95% C.I. 95% C.I.** | |  |
| --- | --- | --- | --- | --- |
|  |  | **Lower** | **Upper** | **P Value** |
| NONFRAILCFStreatedhigh | .314 | .083 | 1.184 | .087 |
| age | 1.084 | .988 | 1.190 | .089 |
| sex | .418 | .123 | 1.423 | .163 |
| CHRchronic | .875 | .598 | 1.280 | .493 |
| MDpolypharmacy | 4.882 | 1.037 | 22.996 | .045 |
| MOCASum | 1.018 | .880 | 1.178 | .812 |
| LOWEDU | .973 | .297 | 3.185 | .964 |
| CLASSICOH | .465 | .056 | 3.863 | .478 |
| Constant | .000 |  |  | .032 |

**Outcome 5 : Deceased by Wave 2**

**(i) Frail by CFS treated low – Basic Logistic Regression Model**

|  | | **Odds Ratio** | **95% C.I. 95% C.I.** | |  |
| --- | --- | --- | --- | --- | --- |
|  |  |  | **Lower** | **Upper** | **P Value** |
|  | FRAILCFStreatedlow | 1.833 | .902 | 3.726 | .094 |
|  | age | 1.105 | 1.071 | 1.141 | .000 |
|  | sex | .654 | .423 | 1.010 | .055 |
|  | Constant | .000 |  |  | .000 |

**(ii) Frail by CFS treated low – Full Binary Logistic Regression Model**

|  | | **Odds Ratio** | **95% C.I. 95% C.I.** | |  |
| --- | --- | --- | --- | --- | --- |
|  |  |  | **Lower** | **Upper** | **P Value** |
|  | FRAILCFStreatedlow | 1.560 | .722 | 3.370 | .258 |
|  | age | 1.090 | 1.042 | 1.140 | .000 |
|  | sex | .826 | .465 | 1.468 | .515 |
|  | CHRchronic | 1.139 | .947 | 1.371 | .168 |
|  | MDpolypharmacy | 1.696 | .900 | 3.194 | .102 |
|  | MOCASum | .888 | .830 | .950 | .001 |
|  | LOWEDU | .673 | .362 | 1.252 | .211 |
|  | CLASSICOH | .890 | .361 | 2.198 | .801 |
|  | Constant | .001 |  |  | .001 |

**(iii) Frail by CFS treated high – Basic Regression Model**

|  | | **Odds Ratio** | **95% C.I. 95% C.I.** | |  |
| --- | --- | --- | --- | --- | --- |
|  |  |  | **Lower** | **Upper** | **P Value** |
|  | FRAILCFStreatedhigh | 1.317 | .723 | 2.402 | .368 |
|  | age | 1.105 | 1.070 | 1.140 | .000 |
|  | sex | .658 | .426 | 1.015 | .058 |
|  | Constant | .000 |  |  | .000 |

**(iv) Frail by CFS treated high – Full Binary Logistic Regression Model**

|  | | **Odds Ratio** | **95% C.I. 95% C.I.** | |  |
| --- | --- | --- | --- | --- | --- |
|  |  |  | **Lower** | **Upper** | **P Value** |
|  | FRAILCFStreatedhigh | .920 | .445 | 1.898 | .821 |
|  | age | 1.093 | 1.044 | 1.143 | .000 |
|  | sex | .828 | .466 | 1.469 | .518 |
|  | CHRchronic | 1.157 | .962 | 1.393 | .122 |
|  | MDpolypharmacy | 1.768 | .944 | 3.312 | .075 |
|  | MOCASum | .885 | .827 | .947 | .000 |
|  | LOWEDU | .656 | .353 | 1.219 | .182 |
|  | CLASSICOH | .879 | .355 | 2.174 | .779 |
|  | Constant | .001 |  |  | .001 |

**(v) Non-frail by CFS treated low – Basic Logistic Regression Mode**

|  | | **Odds Ratio** | **95% C.I. 95% C.I.** | |  |
| --- | --- | --- | --- | --- | --- |
|  |  |  | **Lower** | **Upper** | **P Value** |
|  | NONFRAILCFStreatedlow | .882 | .470 | 1.657 | .697 |
|  | age | 1.135 | 1.097 | 1.173 | .000 |
|  | sex | .844 | .521 | 1.366 | .490 |
|  | Constant | .000 |  |  | .000 |

**(vi) Non-frail by CFS treated low- Full Binary Logistic Regression Model**

|  | | **Odds Ratio** | **95% C.I. 95% C.I.** | |  |
| --- | --- | --- | --- | --- | --- |
|  |  |  | **Lower** | **Upper** | **P Value** |
|  | NONFRAILCFStreatedlow | .923 | .468 | 1.821 | .817 |
|  | age | 1.091 | 1.043 | 1.142 | .000 |
|  | sex | .825 | .465 | 1.466 | .512 |
|  | CHRchronic | 1.153 | .959 | 1.386 | .129 |
|  | MDpolypharmacy | 1.769 | .944 | 3.315 | .075 |
|  | MOCASum | .886 | .828 | .947 | .000 |
|  | LOWEDU | .656 | .353 | 1.220 | .183 |
|  | CLASSICOH | .864 | .349 | 2.138 | .751 |
|  | Constant | .001 |  |  | .001 |

**(vii) Non-frail by CFS treated high – Basic Logistic Regression Model**

|  | | **Odds Ratio** | **95% C.I. 95% C.I.** | |  |
| --- | --- | --- | --- | --- | --- |
|  |  |  | **Lower** | **Upper** | **P Value** |
|  | NONFRAILCFStreatedhigh | .538 | .316 | .914 | .022 |
|  | age | 1.128 | 1.091 | 1.167 | .000 |
|  | sex | .822 | .507 | 1.333 | .426 |
|  | Constant | .000 |  |  | .000 |

**(viii) Non-frail by CFS treated high – Full Binary Logistic Regression Model**

|  | | **Odds Ratio** | **95% C.I. 95% C.I** | |  |
| --- | --- | --- | --- | --- | --- |
|  |  |  | **Lower** | **Upper** | **P Value** |
|  | NONFRAILCFStreatedhigh | .887 | .489 | 1.609 | .693 |
|  | age | 1.092 | 1.044 | 1.142 | .000 |
|  | sex | .829 | .467 | 1.472 | .523 |
|  | CHRchronic | 1.148 | .953 | 1.382 | .146 |
|  | MDpolypharmacy | 1.746 | .930 | 3.281 | .083 |
|  | MOCASum | .888 | .830 | .951 | .001 |
|  | LOWEDU | .663 | .356 | 1.232 | .193 |
|  | CLASSICOH | .877 | .355 | 2.167 | .777 |
|  | Constant | .001 |  |  | .001 |

**Outcome 6 : Any falls or fracture by Wave 2**

**(i) Frail by CFS treated low – Basic Logistic Regression Model**

|  | | **Odds Ratio** | **95% C.I. 95% C.I.** | |  |
| --- | --- | --- | --- | --- | --- |
|  |  |  | **Lower** | **Upper** | **P Value** |
|  | FRAILCFStreatedlow | 1.498 | .951 | 2.358 | .081 |
|  | age | 1.027 | 1.008 | 1.045 | .004 |
|  | sex | 1.664 | 1.325 | 2.088 | .000 |
|  | Constant | .026 |  |  | .000 |

**(ii) Frail by CFS – Full Binary Logistic Regression Model**

|  | | **Odds Ratio** | **95% C.I. 95% C.I.** | |  |
| --- | --- | --- | --- | --- | --- |
|  |  |  | **Lower** | **Upper** | **P Value** |
|  | FRAILCFStreatedlow | 1.185 | .734 | 1.912 | .487 |
|  | age | 1.016 | .994 | 1.038 | .167 |
|  | sex | 1.509 | 1.156 | 1.970 | .002 |
|  | CHRchronic | 1.208 | 1.101 | 1.325 | .000 |
|  | MDpolypharmacy | 1.088 | .826 | 1.434 | .547 |
|  | MOCASum | .983 | .948 | 1.018 | .336 |
|  | LOWEDU | .832 | .625 | 1.108 | .208 |
|  | CLASSICOH | 1.691 | 1.152 | 2.482 | .007 |
|  | Constant | .060 |  |  | .009 |

**(iii) Frail by CFS treated high – Basic Logistic Regression Model**

|  | | **Odds Ratio** | **95% C.I. 95% C.I.** | |  |
| --- | --- | --- | --- | --- | --- |
|  |  |  | **Lower** | **Upper** | **P Value** |
|  | FRAILCFStreatedhigh | 1.646 | 1.160 | 2.334 | .005 |
|  | age | 1.024 | 1.006 | 1.043 | .010 |
|  | sex | 1.682 | 1.340 | 2.112 | .000 |
|  | Constant | .031 |  |  | .000 |

**(iv) Frail by CFS treated high – Full Binary Logistic Regression Model**

|  | | **Odds Ratio** | **95% C.I. 95% C.I.** | |  |
| --- | --- | --- | --- | --- | --- |
|  |  |  | **Lower** | **Upper** | **P Value** |
|  | FRAILCFStreatedhigh | 1.493 | 1.028 | 2.168 | .035 |
|  | age | 1.014 | .991 | 1.036 | .235 |
|  | sex | 1.522 | 1.165 | 1.987 | .002 |
|  | CHRchronic | 1.205 | 1.098 | 1.322 | .000 |
|  | MDpolypharmacy | 1.080 | .820 | 1.421 | .584 |
|  | MOCASum | .986 | .951 | 1.022 | .449 |
|  | LOWEDU | .823 | .618 | 1.095 | .181 |
|  | CLASSICOH | 1.629 | 1.107 | 2.397 | .013 |
|  | Constant | .063 |  |  | .010 |

(**v) Non-frail by CFS treated low –Basic Logistic Regression Model**

|  | | **Odds Ratio** | **95% C.I. 95% C.I.** | |  |
| --- | --- | --- | --- | --- | --- |
|  |  |  | **Lower** | **Upper** | **P Value** |
|  | NONFRAILCFStreatedlow | .771 | .572 | 1.041 | .090 |
|  | age | 1.021 | 1.002 | 1.041 | .028 |
|  | sex | 1.611 | 1.257 | 2.065 | .000 |
|  | Constant | .047 |  |  | .000 |

**(vi) Non-frail by CFS treated low- Full Binary Logistic Regression Model**

|  | | **Odds Ratio** | **95% C.I. 95% C.I.** | |  |
| --- | --- | --- | --- | --- | --- |
|  |  |  | **Lower** | **Upper** | **P Value** |
|  | NONFRAILCFStreatedlow | .804 | .590 | 1.094 | .165 |
|  | age | 1.015 | .992 | 1.037 | .197 |
|  | sex | 1.513 | 1.159 | 1.975 | .002 |
|  | CHRchronic | 1.209 | 1.102 | 1.326 | .000 |
|  | MDpolypharmacy | 1.112 | .846 | 1.463 | .447 |
|  | MOCASum | .981 | .947 | 1.017 | .290 |
|  | LOWEDU | .828 | .622 | 1.102 | .196 |
|  | CLASSICOH | 1.637 | 1.113 | 2.409 | .012 |
|  | Constant | .072 |  |  | .014 |

**(vii) Non-frail by CFS treated high – Basic Regression Model**

|  | | **Odds Ratio** | **95% C.I. 95% C.I.** | |  |
| --- | --- | --- | --- | --- | --- |
|  |  |  | **Lower** | **Upper** | **P Value** |
|  | NONFRAILCFStreatedhigh | .829 | .646 | 1.063 | .139 |
|  | age | 1.021 | 1.002 | 1.041 | .031 |
|  | sex | 1.604 | 1.251 | 2.056 | .000 |
|  | Constant | .049 |  |  | .000 |

**(viii) Non-frail by CFS treated high – Full Binary Logistic Regression Model**

|  | | **Odds Ratio** | **95% C.I. 95% C.I.** | |  |
| --- | --- | --- | --- | --- | --- |
|  |  |  | **Lower** | **Upper** | **P Value** |
|  | NONFRAILCFStreatedhigh | .918 | .700 | 1.203 | .535 |
|  | age | 1.016 | .994 | 1.039 | .160 |
|  | sex | 1.512 | 1.158 | 1.973 | .002 |
|  | CHRchronic | 1.209 | 1.103 | 1.327 | .000 |
|  | MDpolypharmacy | 1.086 | .823 | 1.432 | .560 |
|  | MOCASum | .983 | .949 | 1.019 | .360 |
|  | LOWEDU | .829 | .623 | 1.103 | .198 |
|  | CLASSICOH | 1.695 | 1.154 | 2.489 | .007 |
|  | Constant | .062 |  |  | .009 |

**Outcome 7 : TIA or Stroke by Wave 2**

**(i) Frail by CFS treated low – Basic Regression Model**

|  | | **Odds Ratio** | **95% C.I. 95% C.I.** | | **P Value** |
| --- | --- | --- | --- | --- | --- |
|  |  |  | **Lower** | **Higher** |  |
|  | FRAILCFStreatedlow | .722 | .658 | 3.050 | .658 |
|  | age | 1.043 | .069 | 1.091 | .069 |
|  | sex | .634 | .141 | 1.164 | .141 |
|  | Constant | .003 | .001 |  | .001 |

**(ii) Frail by CFS treated low – Full Binary Logistic Regression Model**

|  | | **Odds Ratio** | **95% C.I. 95% C.I.** | |  |
| --- | --- | --- | --- | --- | --- |
|  |  |  | **Lower** | **Upper** | **P Value** |
|  | FRAILCFStreatedlow | .535 | .122 | 2.348 | .407 |
|  | age | 1.019 | .964 | 1.078 | .497 |
|  | sex | .475 | .232 | .972 | .042 |
|  | CHRchronic | .987 | .794 | 1.228 | .910 |
|  | MDpolypharmacy | 2.506 | 1.174 | 5.346 | .018 |
|  | MOCASum | .933 | .856 | 1.017 | .117 |
|  | LOWEDU | .798 | .382 | 1.666 | .548 |
|  | CLASSICOH | 3.453 | 1.628 | 7.327 | .001 |
|  | Constant | .058 |  |  | .292 |

**(iii) Frail by CFS treated high – Basic Regression Model**

|  | | **Odds Ratio** | **95% C.I. 95% C.I.** | |  |
| --- | --- | --- | --- | --- | --- |
|  |  |  | **Lower** | **Upper** | **P Value** |
|  | FRAILCFStreatedhigh | 2.331 | 1.113 | 4.880 | .025 |
|  | age | 1.033 | .987 | 1.082 | .158 |
|  | sex | .631 | .343 | 1.160 | .138 |
|  | Constant | .005 |  |  | .002 |

**(iv) Frail by CFS treated high – Full Binary Logistic Regression Model**

|  | | **Odds Ratio** | **95% C.I. 95% C.I.** | |  |
| --- | --- | --- | --- | --- | --- |
|  |  |  | **Lower** | **Upper** | **P Value** |
|  | FRAILCFStreatedhigh | 1.547 | .681 | 3.514 | .298 |
|  | age | 1.015 | .960 | 1.074 | .598 |
|  | sex | .471 | .231 | .963 | .039 |
|  | CHRchronic | .964 | .777 | 1.197 | .742 |
|  | MDpolypharmacy | 2.375 | 1.114 | 5.063 | .025 |
|  | MOCASum | .942 | .864 | 1.026 | .172 |
|  | LOWEDU | .811 | .390 | 1.689 | .576 |
|  | CLASSICOH | 3.332 | 1.562 | 7.105 | .002 |
|  | Constant | .064 |  |  | .310 |

**(v) Non-frail by CFS treated low – Basic Logistic regression Model**

|  | | **Odds Ratio** | **95% C.I. 95% C.I.** | |  |
| --- | --- | --- | --- | --- | --- |
|  |  |  | **Lower** | **Upper** | **P Value** |
|  | NONFRAILCFStreatedlow | .537 | .223 | 1.293 | .166 |
|  | age | 1.035 | .989 | 1.083 | .141 |
|  | sex | .560 | .295 | 1.061 | .075 |
|  | Constant | .007 |  |  | .005 |

**(vi) Non-frail by CFS treated low – Full Binary Logistic Regression Model**

|  | | **Odds Ratio** | **95% C.I. 95% C.I.** | |  |
| --- | --- | --- | --- | --- | --- |
|  |  |  | **Lower** | **Upper** | **P Value** |
|  | NONFRAILCFStreatedlow | .558 | .226 | 1.377 | .206 |
|  | age | 1.016 | .962 | 1.074 | .566 |
|  | sex | .463 | .226 | .950 | .036 |
|  | CHRchronic | .965 | .777 | 1.198 | .747 |
|  | MDpolypharmacy | 2.502 | 1.173 | 5.338 | .018 |
|  | MOCASum | .936 | .860 | 1.020 | .132 |
|  | LOWEDU | .828 | .398 | 1.723 | .613 |
|  | CLASSICOH | 3.375 | 1.590 | 7.163 | .002 |
|  | Constant | .080 |  |  | .347 |

**(vii) Non-frail by CFS treated high – Basic Logistic Regression Model**

|  | | **Odds Ratio** | **95% C.I. 95% C.I.** | |  |
| --- | --- | --- | --- | --- | --- |
|  |  |  | **Lower** | **Upper** | **P Value** |
|  | NONFRAILCFStreatedhigh | .968 | .518 | 1.809 | .919 |
|  | age | 1.037 | .991 | 1.086 | .119 |
|  | sex | .567 | .299 | 1.073 | .081 |
|  | Constant | .005 |  |  | .004 |

**(viii) Non-frail by CFS treated high – Full Binary Logistic Regression Model**

|  | | **Odds Ratio** | **95% C.I. 95% C.I.** | |  |
| --- | --- | --- | --- | --- | --- |
|  |  |  | **Lower** | **Upper** | **P Value** |
|  | NONFRAILCFStreatedhigh | 1.340 | .669 | 2.686 | .409 |
|  | age | 1.020 | .965 | 1.078 | .485 |
|  | sex | .470 | .229 | .963 | .039 |
|  | CHRchronic | .984 | .791 | 1.224 | .884 |
|  | MDpolypharmacy | 2.542 | 1.184 | 5.456 | .017 |
|  | MOCASum | .932 | .854 | 1.016 | .110 |
|  | LOWEDU | .817 | .392 | 1.700 | .588 |
|  | CLASSICOH | 3.497 | 1.649 | 7.417 | .001 |
|  | Constant | .048 |  |  | .259 |
