# Supplementary material for: Do the frail experience more adverse events from intensive blood pressure control? A 2-year prospective study in the Irish Longitudinal Study on Ageing (TILDA)
Source: eClinicalMedicine. 2022 Feb 19;45:101304. doi: 10.1016/j.eclinm.2022.101304 (PMC8860911; doi:10.1016/j.eclinm.2022.101304)
Supplement: Supplementary file 3 [file mmc3.docx]

**Captions for Supplementary Material**

1. Caption for Appendix 1 :

Appendix 1 : Basic Binary Logistic Regression Models and Full Binary Logistic Regression Models for the Frail by Frailty Penotype (FP)- Blood Pressure Groups

2. Caption for Appendix 2 :

Appendix 2 : Basic Binary Logistic Regression Models and Full Binary Logistic Regression Models for the Frail by Clinical Frailty Scale (CFS)- Blood Pressure Groups
